# Gene duplication drives genome expansion in a major lineage of Thaumarchaeota

**DOI:** 10.1038/s41467-020-19132-x

**Published:** 2020-10-30

**Authors:** Paul O. Sheridan, Sebastien Raguideau, Christopher Quince, Jennifer Holden, Lihong Zhang, Thames Consortium, William H. Gaze, Jennifer Holden, Andrew Mead, Sebastien Raguideau, Christopher Quince, Andrew C. Singer, Elizabeth M. H. Wellington, Lihong Zhang, Tom A. Williams, Cécile Gubry-Rangin

**Affiliations:** 1 School of Biological Sciences, University of Aberdeen, Aberdeen, UK; 2 School of Biological Sciences, University of Bristol, Bristol, UK; 3 Warwick Medical School, University of Warwick, Coventry, UK; 4 Organisms and Ecosystems, Earlham Institute, Norwich, UK; 5 Gut Microbes and Health, Quadram Institute, Norwich, UK; 6 School of Life Sciences, University of Warwick, Coventry, UK; 7 European Centre for Environment and Human Health, Medical School, University of Exeter, Exeter, UK; 8 Rothamsted Research, Harpenden, UK; 9 UK Centre for Ecology & Hydrology, Wallingford, OX10 8BB UK

**Keywords:** Phylogenetics, Genome evolution, Archaeal genomics, Metagenomics

## Abstract

Ammonia-oxidising archaea of the phylum Thaumarchaeota are important organisms in the nitrogen cycle, but the mechanisms driving their radiation into diverse ecosystems remain underexplored. Here, existing thaumarchaeotal genomes are complemented with 12 genomes belonging to the previously under-sampled Nitrososphaerales to investigate the impact of lateral gene transfer (LGT), gene duplication and loss across thaumarchaeotal evolution. We reveal a major role for gene duplication in driving genome expansion subsequent to early LGT. In particular, two large LGT events are identified into Nitrososphaerales and the fate of these gene families is highly lineage-specific, being lost in some descendant lineages, but undergoing extensive duplication in others, suggesting niche-specific roles. Notably, some genes involved in carbohydrate transport or coenzyme metabolism were duplicated, likely facilitating niche specialisation in soils and sediments. Overall, our results suggest that LGT followed by gene duplication drives Nitrososphaerales evolution, highlighting a previously under-appreciated mechanism of genome expansion in archaea.

## Abstract (web summary)

Ammonia-oxidising archaea of the phylum Thaumarchaeota are important organisms in the nitrogen cycle. Using 12 new genomes, this study finds evidence that Nitrososphaerales evolution was marked by lateral gene transfer followed by gene duplication.

## Introduction

Thaumarchaeota have attracted much attention since their discovery, with accumulating evidence of their ubiquitous distribution and crucial ecosystem function in many environments^1–3^. Isolation and cultivation demonstrated that many of these organisms derive energy from the oxidation of ammonia under aerobic conditions. Ammonia oxidation is limited to a small number of microbial phyla but is an important microbial process as it implements an essential and rate-limiting step in the global nitrogen cycle, the conversion of ammonia to nitrite (via hydroxylamine). Within Thaumarchaeota, ammonia-oxidising archaea (AOA) are ubiquitous and abundant in soils and oceans. At present, all identified AOA are members of the class Nitrososphaeria^4^ and are classically placed in four order-level phylogenetic lineages^1^: the Nitrososphaerales^5^, Nitrosopumilales^6^, *Candidatus* Nitrosotaleales^7^ and *Candidatus* Nitrosocaldales^8^.

While a substantial number of ecological studies and several cultures and enrichments confirm the ammonia-oxidising activity of Thaumarchaeota, there is also evidence that ammonia oxidation is not universal in this phylum^9–13^. In particular, there is evidence that the early-diverging lineages of Thaumarchaeota (including the Group 1.1c, Group 1.3 and pSL12 lineages) do not require ammonia oxidation for growth^10,13^, and the ammonia oxidation machinery may have been acquired subsequently to the origin of Thaumarchaeota during, or after, the Great Oxygenation Event 2300 My ago^12^. While oxygen and pH have been recognised as driving forces of massive diversification through their evolutionary history^12,14^, the evolutionary mechanisms leading to such high phylogenetic and metabolic diversity have received little attention.

A large lateral gene transfer event in the last common ancestor (LCA) of AOA is proposed to have played a major role in their transition to a chemolithoautotrophic lifestyle^12,15^. However, there is currently little evidence for other cases of large lateral gene acquisition in Thaumarchaeota evolution^16,17^, and little is known of the relative contributions of gene duplications and genes losses. Large-scale events of lateral gene transfer (LGT) have been suggested as major drivers of proteome evolution in diverse archaeal lineages^18^, including Thaumarchaeota^16^. However, the long evolutionary history of Thaumarchaeota (>2.3 billion years of evolution) and their diversification in a range of ecosystems^2,12,14^ raises the possibility that distinct evolutionary mechanisms have shaped thaumarchaeotal genomes across their history. In particular, we hypothesise that an ongoing process of LGT spread across thaumarchaeotal evolution (in contrast, or addition to acquisitions at their origin) was the major source of molecular innovation. Phylogenomic methods based on reconstructed ancestral gene contents have been used previously throughout the archaeal radiation to explicitly model gene family acquisitions, duplications and losses using a recently-developed approach for probabilistic gene mapping^19^ by amalgamated likelihood estimation (ALE)^20^. However, the aforementioned study only included a limited number of Thaumarchaeota. Similarly, a recent comparative analysis of a diverse set of thaumarchaeotal genomes examining the influence of oxygen availability on diversification did not include the large diversity of terrestrial thaumarchaeotal genomes presented here, nor disentangle the contributions of gene transfer and gene duplication to genomic diversification^15^.

Here we present 12 high-quality metagenome-assembled genomes (MAGs), providing the first genome representatives of three of the six known major lineages of the prevalent terrestrial order of AOA, the Nitrososphaerales. This enables the reconstruction of a strongly supported phylogeny for the Nitrososphaerales, which was not previously achieved using single-gene markers. Then, we investigated the mechanisms of genome evolution in the Thaumarchaeota phylum, quantifying the relative contributions of lateral gene transfers, gene duplications and gene losses. Phylogenomic analysis reveals two previously uncharacterised large lateral gene transfer events followed by subsequent extensive gene duplication mechanisms that have shaped the evolution of Nitrososphaerales.

## Results

### Thaumarchaeotal phylogenomic diversity

**Fig. 1:**
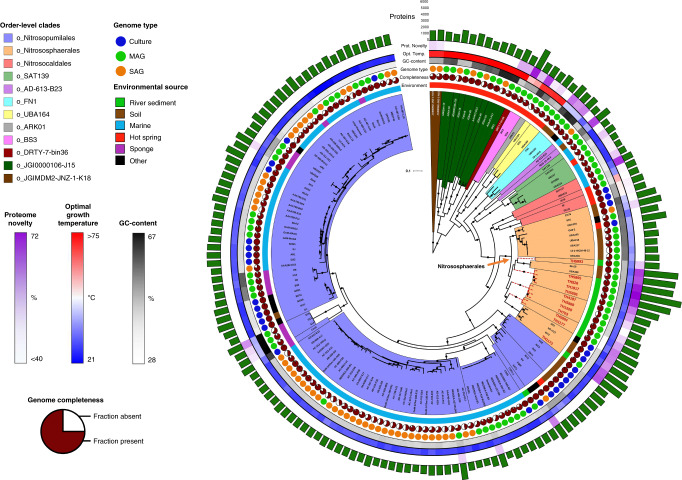
Phylogenomic tree of Thaumarchaeota. This tree includes 152 thaumarchaeotal genomes (29 culture genomes, 51 single-cell-assembled genomes (SAG) and 72 metagenome-assembled genomes (MAGs), including 12 novel MAGs obtained in this study) from a range of environments and 11 Aigarchaeota genomes and is rooted with two Bathyarchaeota genomes. The tree was inferred by maximum likelihood from 75 concatenated phylogenetic markers, which were aligned separately and analysed using the best-fitting model for each alignment. The 12 new genomes assembled in this study are indicated by red tip labels. Clades that previously lacked representative genomes are indicated with dotted red branches. Dots indicate branches with >95% UFBoot and SH-aLRT support. Coloured order-level clades are further divided into family-level clades with black clade borders. Protein novelty is defined as the percentage of encoded proteins that lack a close homologue (e-value <10^−5^, % ID >35, alignment length >80 and bit score >100) in the arCOG database. Detailed genome information is given in Supplementary Data 1.

Twelve thaumarchaeotal genomes of 63–97% completeness (Supplementary Data 1) were reconstructed by large-scale co-assembly of 171 metagenomes from sediment samples collected from 23 sites along the River Thames, UK. Phylogenomic analysis of 75 concatenated single-copy orthologues retrieved from 152 available thaumarchaeotal genomes (including the 12 genomes assembled in this study) (Supplementary Data 1) provided a well-supported thaumarchaeotal phylogenomic tree (Fig. 1), with most nodes with ultrafast bootstrap (UFBoot) values >95% and Shimodaira and Hasegawa-like approximate likelihood ratio test (SH-aLRT) values >95%. This phylogenomic tree was the best-supported tree after comparison of several marker gene selections (SI: Extended phylogenomics). The tree is highly congruent with previously published work^12^, with the exception of several early-diverging lineages (SI: Extended phylogenomics). Previous classifications of Thaumarchaeota have been mainly based on the single marker 16S rRNA gene^5,21–23^ with the exception of a few studies using concatenated protein trees^12,15,24^, while the ammonia monooxygenase (*amo*A) gene was used as a proxy marker for classification of AOA^2,4,14^, as *amo*A gene is not present in all members of the phylum. Classification of non-AOA within Thaumarchaeota is based on monophyletic placement rather than unified metabolism at the phylum level. We therefore suggest a possible thaumarchaeotal taxonomic ranking based on our phylogenomic analysis that maintains maximum consistency with previous work while incorporating the early-diverging lineages that lack *amo*A^2,25,26^ (Supplementary Data 2). Our thaumarchaeotal genome dataset represents a diverse phylum comprising 8 classes (1 AOA, 7 non-AOA), 10 orders (3 AOA, 7 non-AOA), 28 families (18 AOA, 10 non-AOA), 31 genera (20 AOA, 11 non-AOA) and 103 species (87 AOA, 16 non-AOA). While the classically used thaumarchaeotal nomenclature is congruent with the taxonomic stratification, a few exceptions were observed. For example, the order Nitrosopumilales encompasses the formally defined order *Candidatus* Nitrosotaleales^27^, whose classification may now need to be reconsidered. In addition, this taxonomic stratification indicates that Nitrososphaerales contains a minimum of 8 genera, with *Ca*. Nitrososphaera gargensis belonging to a different genus to *Nitrososphaera viennensis* and *Ca*. Nitrososphaera evergladensis.

We also used the Genome Taxonomy Database Toolkit (GTDB-Tk) to evaluate the genomic diversity of our 12 river sediment MAGs (Supplementary Data 3). While one of these genomes (TH1173) is a close relative of a published Nitrososphaerales genome, *Ca*. Nitrosocosmicus oleophilus MY3, the range of relative evolutionary divergence (RED)^28^ values for the other 11 MAGs was 0.72–0.82, suggesting that some of these MAGs represent new family-level lineages (Supplementary Data 3). These results are consistent with our phylogenomic analysis, in which these genomes represent novel deep branches within Nitrososphaerales (Fig. 1). Assessment of the diversity of the genome set revealed that specific lineages are disproportionately represented in terms of genome sequence based on the diversity indicated by *amo*A gene studies (Supplementary Fig. 1), as observed previously^2^. While the Nitrososphaerales genomic diversity is now relatively well represented with the addition of these genomes, several major clades of the Nitrosopumilales lack representative genomes. Previously, broad groups loosely reflecting the origins of thaumarchaeotal isolates and genomes were described as deep-water AOA, shallow-water AOA, terrestrial AOA and basal (non-ammonia-oxidising) Thaumarchaeota^12^. The deep-water AOA group (clade NP-Alpha-2) is the least diverse set of genomes, with 33 genomes affiliating to a single genus (max. divergence 26%), closely followed by the shallow-water AOA group (clades NP-Epsilon-2; NP-Gamma-2; NP-Theta-4; NP-Delta-2), with 62 genomes forming 4 genera (max. divergence 44%) (Supplementary Fig. 2). The terrestrial AOA group (clades NP-Eta, NT-Alpha and NS) represent the most diverse AOA set of genomes, with 40 genomes forming 15 genera (max. divergence 48%). However, the basal thaumarchaeotal group appears to be even more diverse with 17 genomes forming 11 genera (max. divergence 57%) (Supplementary Fig. 2).

The genomes used in this study presented relatively high completeness (average of 82%) with lowest values being reported for single-cell genomes from the marine environment (Fig. 1), low contamination (average of 1%) and variable strain heterogeneity (average of 34%) (Supplementary Data 1). Despite the wide range of genome size across the phylum (0.8–5.2 Mbp), the data clearly support higher genome sizes in some of the terrestrial Nitrososphaerales. This higher genome size was noted previously in several cultured Nitrososphaerales^29^ and is particularly pronounced in the MAGs within the genera g_Bin19 and g_TH5895. In agreement with the findings of Alves et al.^2^, GC content is closely related to phylogeny in most cases, with a notable exception being the sponge-associated Thaumarchaeota, which present a higher GC content, potentially due to adaptation to environmental stresses such as nutrient and energy limitation^30^. Prediction of optimal growth temperatures (OGTs) confirmed that most of the Nitrososphaerales and Nitrosopumilales are mesophilic with OGTs between 21 and 37 °C, with a few exceptions such as organisms affiliating to three closely related terrestrial genera (g_EN76, g_UBA210 and g_Nitrososphaera) or to two genera containing exclusively sponge-associated Thaumarchaeota (g_Cenarchaeum and g_S14) with slightly higher temperature optimum (range 33–42 °C) (see SI: Extended optimal growth temperature). As predicted, Nitrosocaldes have higher OGTs (range 32–56 °C), but these estimates are lower than measured OGT in the two cultures available (difference of 15 and 19 °C, SI: Extended optimal growth temperature). Predicted OGTs among the deeply-rooted non-AOA Thaumarchaeota are consistent with the hypothesis that Thaumarchaeota evolved from a hyperthermophilic common ancestor (Fig. 1 and Supplementary Data 1), but the presence of the marine mesophiles SAT137 and UBA213 in Nitrosocaldales (both sharing 84% amino acid identity on the *amo*A gene with the thermophilic *Ca*. Nitrosocaldus islandicus 3F) and mesophiles in their closest non-AOA relatives (o_FN1, o_AD-613-B23 and o_SAT139) are consistent with the hypothesis that the last common ancestor of AOA was a mesophile, placing the hot origin of Thaumarchaeota further back in evolutionary history than previously proposed^15,23^.

### Phylogeny and metabolic traits of the Nitrososphaerales lineage

**Fig. 2:**
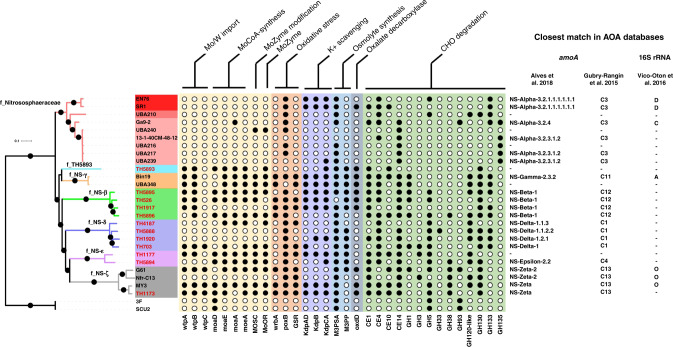
Phylogeny and distinctive metabolic traits of Nitrososphaerales. This tree includes 26 Nitrososphaerales genomes and was inferred by maximum likelihood reconstruction from 188 concatenated marker genes. The 12 new genomes obtained in this study are indicated by red tip labels and the tree is rooted with the *Ca*. Nitrosocaldales strains 3F and SCU2. This tree has similar topology to the phylum-wide tree, but with strong support for almost all branches. Dots indicate branches with >95% UFBoot and SH-aLRT support. Colours on branches and leaf labels indicate families and genera, respectively. While many of the genes acquired in early stages of Nitrososphaerales evolution are uncharacterised, several genes involved in molybdoenzymes synthesis and stress response could be identified, among which several are represented on the figure. The presence and absence of those genes are indicated by a filled or empty circle, respectively. The following genes were used in the figure: *wtpABC*, Wtp transport system subunits A, B and C; MOSC, molybdenum cofactor sulfurase; MoOR, Molybdopterin oxidoreductase; *wrbA*, NAD(P)H quinone oxidoreductase; *poxB*, pyruvate oxidase; GSR, glutathione-disulfide reductase; *kdpABC*, potassium transport system subunits A, B and C; M3PSA, mannosyl-3-phosphoglycerate synthase; M3PP, mannosyl-3-phosphoglycerate phosphatase; CE, carbohydrate esterase family; GH, glycoside hydrolase family. Closest BLASTn^62^ matches in the Alves^2^, Gubry-Rangin^14^ and Vico-Oton^21^ databases are indicated for the Nitrososphaerales genomes on the right of the figure. Genomes in which *amoA* or 16S rRNA genes were not detected are indicated with a hyphen (-).

The 12 MAGs reconstructed in this study represent the first genome representatives of three of the six Nitrososphaerales families, enabling a detailed analysis of this order. As the whole-phylum analysis did not result in a highly supported phylogeny of the Nitrososphaerales order, a more targeted phylogenomic analysis based on 188 Nitrososphaerales markers was performed to increase resolution (Fig. 2). Previously available genomes and the new MAGs represent 22 species and 9 genera within Nitrososphaerales. The basal tree topologies of this order based on the widely-used single-gene marker *amo*A^2,14^ or on 188 single-copy core markers are different, but a phylogenomically-informed rooting of the *amo*A phylogeny resolved many of the incongruencies (Supplementary Fig. 3). This phylogenomic approach also provides robust resolution (>95% ultrafast bootstrapping) of the basal relationship between the lineages NS-alpha, NS-beta and NS-gamma, which was not obtained in previous studies using the *amo*A phylogeny alone. Moreover, the genome TH5893 appears to represent a previously undiscovered seventh family of Nitrososphaerales, but this remains unclear as its relationship to NS-gamma was not resolved with high confidence (Fig. 2) and *amo*A and 16S rRNA genes were not detected in this MAG. The genomes in this work represent the known family-level diversity of Nitrososphaerales, but many of the lower taxonomic ranks identified in *amo*A-based studies have no genome sequenced. Despite the greater statistical power of the phylogenomic data compared to the *amo*A gene approach, the limited diversity coverage may have potential phylogenetic biases and future increased genome representation in these ranks may improve phylogeny prediction in this group.

AOA are a highly metabolically conserved group of chemolithoautotrophic organisms, which use ammonia for energy^31^ and fix CO_2_ as a carbon source^32^. They also have the capacity to synthesise many of the cofactors^33^ and amino acids^29^ required for their cellular function. Despite this high level of metabolic conservation, Nitrososphaerales AOA possess additional metabolic capacities that likely facilitated their expansion into a wide variety of soil, wastewater and river sediment environments^34^ (Fig. 2). Among the traits acquired early in the Nitrososphaerales diversification, many likely contribute to protection against osmotic and oxidative stress, production of molybdoenzymes and carbohydrate utilisation (Supplementary Data 7).

Carbohydrate active enzymes (CAZymes) are ubiquitous in the tree of life, allowing organisms to degrade environmental carbohydrates as energy and carbon sources (such as the utilisation of monosaccharides from cellulose or hemicellulose degradation) and for the formation of more complex compounds from simpler substrates (such as extracellular polysaccharide synthesis)^35^. The number of CAZyme genes in the Nitrososphaerales lineage is large and greater than that of other members of the phylum, as the other Thaumarchaeota encode an average of less than two CAZymes. The last common ancestor of the Nitrososphaerales is predicted to have acquired the amylo-α-1,6-glucosidase GH133 (EC3.2.1.33) and polyspecific carbohydrate esterases CE1, CE4 and CE10. In addition, the beta-mannanase GH130, alpha-mannanase GH38, hemicellulases GH1, GH3 and GH5, and a putative chitin disaccharide deacetylase (CE14) were also acquired during Nitrososphaerales diversification.

Since these organisms lack the phosphofructokinase necessary to complete the glycolytic pathway, it is difficult to determine the purpose of these carbohydrate degrading enzymes. One possible explanation is that the resulting monosaccharides are being utilised for the biosynthesis of cellular components such as extracellular polysaccharides or osmolytes. Indeed, the Nitrososphaerales possess mannosyl-3-phosphoglycerate synthase and mannosyl-3-phosphoglycerate phosphatase genes, giving these organisms the potential ability to synthesise the osmoprotectant mannosyl-glycerate from mannose monosaccharides and glycerate 3-phosphate. They have also lost the mannose-6-phosphate isomerase that is present in most other Thaumarchaeota, preventing mannose monosaccharides being shunted into early glycolysis, increasing the amount of mannose available for mannosyl-glycerate synthesis.

Kdp, a high-affinity ATP-driven potassium uptake system that enables K^+^-mediated osmoregulation in potassium limited environments^36^, is present in several Nitrososphaerales genomes, but almost completely absent from marine Thaumarchaeota (with the exception of REDSEA-S19-B12N3). In the event of osmotic shock, this system could allow the organisms to maintain turgor pressure in soils where there is low potassium concentration or where potassium is bound to negatively charged humus or clay particles by cation exchange.

Nitrososphaerales have also acquired several systems for protection against oxidative stress indicating that there may be additional production of reactive oxygen species (ROS) in the metabolism of this lineage. These include pyruvate oxidase (*PoxB*), NAD(P)H quinone oxidoreductase (*WrbA*) and glutathione-disulfide reductase (*GSR*). *PoxB* catalyses the ubiquinone-dependent oxidative decarboxylation of pyruvate to form acetate and CO_2_. This pathway enables the production of acetate in a NAD-independent manner, preventing the accumulation of ROS from resulting NAD+ regeneration by NADH dehydrogenase in slow-growing cells where ROS are not diluted by cell growth^37^. *WrbA* maintains quinones in the fully reduced state, guarding against the production of ROS from one-electron redox cycling^38^ and *GSR* catalyses the reduction of glutathione disulfide to two reduced molecules of glutathione—an antioxidant that protects cellular components from oxidative stress.

Another notable difference between the Nitrososphaerales and other lineages of AOA is the presence of genes for the uptake of molybdate and synthesis of molybdoenzymes (MoZymes). These transport and synthesis genes were previously detected in three *Ca*. Nitrosocosmicus strains^39^ and appear to be also present in several of the newly represented Nitrososphaerales lineages (Fig. 2). The *Wtp* transport system, which has high affinity for both molybdate and tungstate, is present in many Nitrososphaerales, but almost completely absent in other Thaumarchaeota (with the exception of the non-AOA Thaumarchaeota ARK01, UBA164 and YP1-bin3). The enzymes *MoaA*, *MoaD/E* and *MoeA* catalyse the formation of molybdenum cofactor (MoCo) of MoZymes and MOSC transfers sulfur to the molybdenum, forming mono-oxo MoCo^40^. MoZymes of the sulfite oxidase (SO) superfamily were identified in several members of the Nitrososphaerales order (Supplementary Fig. 4). These enzymes appear to be an intermediate between the eukaryotic SO and the bacterial protein-methionine-sulfoxide reductase (Supplementary Fig. 4). The majority of the Nitrososphaerales MoZymes are structurally similar to the eukaryotic SO, possessing both the oxidoreductase molybdopterin binding domain (PF00174) and the MoCo oxidoreductase dimerisation domain (SSF81296), but lacking the N-terminal cytochromes b5 electron transport hemoprotein domain (PF00173). These enzymes, however, possess at their N-terminal several transmembrane helicases that are likely to be a novel class of cytochrome domains. We would therefore hypothesise that this protein family is a class of archaeal SO that convert sulfite to sulfate, allowing the generation of ATP in oxidative phosphorylation. This reaction produces hydrogen peroxide as a by-product^41^, possibly explaining the need for extra oxidative stress protection mechanisms in this order.

The three MoZymes of the artic soil MAG, Bin19, and one of the two MoZymes of *Ca*. Nitrosocosmicus oleophilus (MY3_01316) were more similar in structure and sequence to the bacterial protein-methionine-sulfoxide reductase that repairs oxidized periplasmic proteins containing methionine sulfoxide residues than to the eukaryotic sulfite oxidases, indicating a putative role of this enzyme in oxidative stress damage protection.

### Influence of evolutionary mechanisms on thaumarchaeotal diversification

**Fig. 3:**
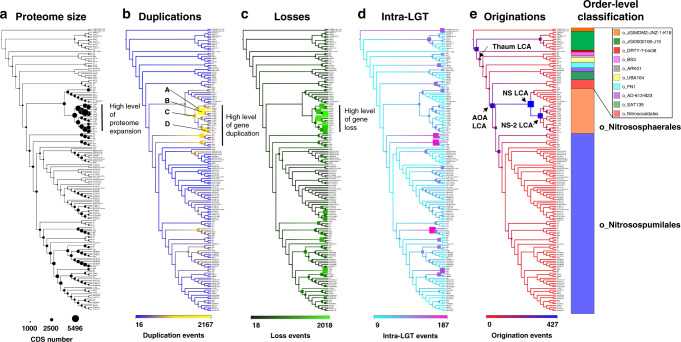
Quantified mechanisms of proteome evolution. The quantitative and qualitative predictions of the proteome changes were estimated across the thaumarchaeotal history and reported on the thaumarchaeotal cladogram possessing the topology of the ML tree presented in Fig. 1. In addition to the changes in proteome size (panel **a**), four mechanisms of proteome changes were quantified: duplication (panel **b**; copying of a gene within a genome), loss (panel **c**; loss of a gene within a genome), intra-LGT (panel **d**; defined as the acquisition of a gene from other member(s) of the phylum) and origination (panel **e**; defined as the acquisition of a gene from members of phyla outside the sampled genome set (Inter-LGT) or by de novo gene formation). For each cladogram, scale numbers indicate the range of the predicted number of events for a given mechanism. The number of events occurring on each branch is represented by the colour of the associated boxes using the colour legend at the bottom of the cladogram and box sizes increase relatively to the number of events. The order-level classification of the genomes is indicated by the coloured bar on the right. Duplication and loss hotspots within the Nitrososphaerales were highlighted by vertical bars in panels (**b**) and (**c**) and letters A, B, C (which corresponds to NS-2 LCA) and D indicate branches with extensive duplication. Originations occurring in the last common ancestors of all Thaumarchaeota, all ammonia-oxidising archaea, all Nitrososphaerales and a multifamily sub-group within Nitrososphaerales are labelled Thaum LCA, AOA LCA, NS LCA and NS-2 LCA, respectively.

The exhaustive creation of probabilistic ancestral reconstructions from every branch of the thaumarchaeotal phylogeny allowed the characterisation and quantification of proteome changes along every lineage (Fig. 3 and Supplementary Fig. 5; Supplementary Data 4). The majority of the predicted 67,400 gene content gains in Thaumarchaeota evolution occurred through 51,653 duplications (77% of gains) of pre-existing genes, with 11,430 intra-phylum gene transfers (intra-LGT) (17% of gains) and 4317 originations (including inter-phyla gene transfers (inter-LGT) and de novo gene formation) (6% of gains). There were also 227,837 gene losses predicted, indicating gene duplication and gene loss as the two most significant drivers of gene content change in Thaumarchaeota evolution. The same ancestral genes were lost along multiple lineages, explaining the higher number (nearly 3.5-fold) of losses than gains.

Four branches within the Nitrososphaerales order (noted A, B, C and D in Fig. 3, panel b) are estimated to undergo particularly high rates of gene duplication, collectively accounting for 11% of duplication events in the phylum (Fig. 3, Supplementary Fig. 5 and Supplementary Data 4), with more than 900 duplication events occurring on each of those branches. Numerically, these are the major mechanisms of predicted proteome expansion in the Thaumarchaeota phylum, resulting in most Nitrososphaerales encoding more than 3000 proteins and possessing an average genome size of 2.7 Mbp (range 1.5–5.2 Mbp). Duplication of existing genes also appears to be the major mechanism of predicted proteome expansion in the *Ca*. Nitrosotalea and *Ca*. Nitrosotenuis genera with their last common ancestors undergoing 1337 and 1010 duplication events, respectively (Supplementary Data 4). This resulted in the extant members of these genera possessing the largest proteomes in the Nitrosopumilales order.

These duplication hotspots resulted in the expansion of several arCOG families (Supplementary Data 5), with copy numbers increasing more than 10-fold in families predicted to be involved in transcription (arCOG04362, arCOG01760 and arCOG01055), signal transduction (arCOG02391), carbohydrate transport (arCOG00144) and coenzyme metabolism (arCOG00972) (Supplementary Data 5). In parallel to these expansions, the copy number of other arCOGs decreased (Supplementary Data 6). Interestingly, the three most notable contractions in NS-beta LCA (arCOG01067, NADH dehydrogenase; arCOG04946, lactoylglutathione lyase-like enzyme and arCOG00041, phosphoribosyltransferase) (Supplementary Data 6) actually increased in copy number in its ancestor (NS-2 LCA) (Supplementary Data 5). This indicates a fluctuating expansion and contraction of families throughout evolutionary history, rather than a consistent selective direction.

The evolution of the Nitrososphaerales lineage appears to have involved a significantly larger number of proteome changes than the phylum as a whole (*P* < 1e−6), with greater numbers of duplications (*P* < 0.006) and losses (*P* < 5e−5) explaining much of this difference.

Although duplications are the major mechanism of proteome expansion in Thaumarchaeota, acquisition of a large number of gene families from outside the phylum have also largely contributed. This is particularly true for the LCA of ammonia-oxidising Thaumarchaeota (AOA LCA), where the origination of 320 gene families contributed to the transition to a chemolithoautotrophic ammonia-oxidising lifestyle (Fig. 3 and Supplementary Data 4). Such large gene family gains in AOA LCA have also been predicted in earlier studies^12,15^. All major origination events (182–427 gene families) have occurred early in the evolutionary history of ammonia-oxidising Thaumarchaeota and accounted for 48% of all gene family originations in the class Nitrososphaeria. In addition, large gene family origination (166 gene families) also occurred in the last common ancestor of the entire thaumarchaeotal phylum (Thaum LCA in Fig. 3).

**Fig. 4:**
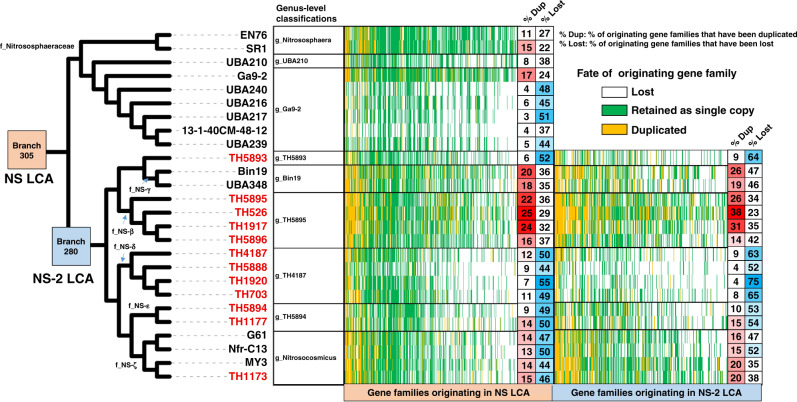
Fate of the originating gene families in Nitrososphaerales lineages: evidence for subsequent extensive duplication and loss. The cladogram highlights two branches that experienced large origination events, NS LCA and NS-2 LCA. The originating gene families are extensively duplicated in some lineages, such as g_TH5895, but also extensively lost in other lineages, such as g_TH4187. The ‘% Dup’ and ‘% Lost’ values have been corrected for genome completeness. The 12 new genomes assembled in this study are indicated by red tip labels. Genus-level taxonomic affiliations are presented next to the cladogram tips.

Two large origination events were predicted in the Nitrososphaerales introducing 427 and 353 new gene families into the last common ancestor of Nitrososphaerales (NS LCA) and an early descendant of NS LCA (NS-2 LCA) (Fig. 4), making these the two largest origination events detected in the phylum (Fig. 3). The majority of the genes originating on these branches (71 and 83% in NS LCA and NS-2 LCA, respectively, Supplementary Data 7) lack close matches in the arCOG database^12^, reflecting a high level of proteome novelty (Fig. 1). The source of these originating gene families may be inter-LGT or de novo gene formation. Homologues of the majority of these gene families (84 and 59% in NS LCA and NS-2 LCA, respectively, Supplementary Data 8) were detected in a larger database of archaea, bacteria and eukaryote genomes, indicating their lateral acquisition. Most gene families acquired by NS LCA (Fig. 3) are predicted to have been mainly transferred laterally from other archaea (archaea 95%, bacteria 5%) (Supplementary Data 8), whereas gene families acquired in NS-2 LCA (Fig. 3) were predicted to have been transferred from a variety of archaea (64%), bacteria (34%) and eukaryotes (2%) (Supplementary Data 8). Gene transfer from other archaea was also previously demonstrated in the sister phylum Aigarchaeota^15^.

These originating gene families encountered different fates in the different lineages of Nitrososphaerales (Fig. 4), with some families being subject to duplications or losses. Interestingly, half of originating gene families (47 and 54% for NS LCA and NS-2 LCA, respectively) were duplicated within at least one extant member of this order (Fig. 4). The combination of these gene family originations followed by intensive duplications have resulted in members of the Nitrososphaerales having among the highest levels of proteome novelty in Thaumarchaeota (Fig. 1). A large number of losses (up to 75% of the originating gene families) occurred in g_TH4187 and g_Ga9-2 (Fig. 4). Conversely, there was intensive duplication of originating gene families (up to 29% of the originating gene families) in the g_TH5895 and g_Bin19, the genera with the largest genomes in the phylum, indicating that originating genes are duplicated rather than lost in case of proteome expansion. Similar trends of extensive loss in g_TH4187 and g_Ga9-2, and duplication in g_TH5895 and g_Bin19 were observed in ancestral gene families (originating prior to the LCA of Nitrososphaerales) (Supplementary Data 9), but were significantly higher for originating gene families in g_TH4187 (*P* < 0.01), g_Ga9-2 (*P* < 0.01) and g_TH5895 (*P* < 0.05). The lineage-specific nature of gene duplication and loss of these originating gene families also suggests that they may be more important for specialisation of the different Nitrososphaerales lineages into distinct ecological niches than for more general soil and sediment environmental colonisation.

### Major transitions in thaumarcheotal evolution

Thaumarchaeota proteomes have undergone extensive gene content changes during their evolution alongside important environmental niche transitions; this has resulted in a highly metabolically diverse phylum. The nature and influence of these gene content changes were analysed through ancestral reconstruction of the metabolic pathways at key nodes in the thaumarchaeotal phylogeny (Supplementary Data 10).

The most extreme transition in the thaumarchaeotal history was probably the transition from the thaumarchaeotal LCA (Thaum LCA) to the ammonia-oxidising archaeal LCA (AOA LCA). Based on the genomic predictions, Thaum LCA was a heterotrophic hyperthermophilic organism utilising carbohydrates as energy and carbon sources, with a complete glycolytic pathway (Supplementary Data 10). The absence of genes for biosynthesis of cobalamin, riboflavin and biotin indicate that it acquired vitamins from extracellular organic sources. The transition from the Thaum LCA to AOA LCA was accompanied by several functional gain and loss events (Supplementary Data 11 and 12). Most notably, this transition involved the acquisition of the ammonia monooxygenase genes (*amoABC*), which generate energy by oxidising ammonia to hydroxylamine. This event coincided with the acquisition of urease (EC:3.5.1.5), which converts urea to ammonia, and genes for the biosynthesis of cobalamin, riboflavin and biotin. AOA LCA also acquired the ability to biosynthesise asparagine, alanine and threonine from aspartate through the acquisition of aspartate 4-decarboxylase (EC:4.1.1.12), asparagine synthase (EC:6.3.5.4), aspartate kinase (EC:2.7.2.4) and threonine synthase (EC:4.2.3.1) (Supplementary Data 11 and 12).

The evolutionary transition from Thaum LCA to the ammonia-oxidising lifestyle of AOA LCA involved the loss of glucokinase (EC:2.7.1.2) and phosphofructokinase (EC:2.7.1.11), preventing them from generating ATP by glycolysis. They also lost the glycolysis-associated carbohydrate degrading enzymes alpha-N-arabinofuranosidase (EC:3.2.1.55), alpha-amylase (EC:3.2.1.1), alpha-mannosidase (EC:3.2.1.24) and the CO_2_ fixing genes phosphoenolpyruvate carboxylase (EC:4.1.1.31) and pyruvate ferredoxin oxidoreductase (EC:1.2.7.1). These losses were concomitant with the acquisition of the 3-hydropropionate/4-hydroxybutrate CO_2_ fixation pathway genes methylmalonyl-CoA epimerase (EC:5.1.99.1) and 4-hydroxybutyryl-CoA dehydratase (EC:4.2.1.120) (Supplementary Data 11 and 12)^32^.

The establishment of AOA LCA as a chemolithoautotroph was followed by diversification into three order-level groups: Nitrosocaldales, present in hot spring and marine environment; Nitrososphaerales, present mostly in river sediments and soil; and Nitrosopumilales present mostly in marine, soil and sponge-associated environments.

The transition from AOA LCA to the last common ancestor of Nitrosocaldales (NC LCA) involved several functional gains, including geranylgeranylglycerol-phosphate geranylgeranyltransferase (EC:2.5.1.42), which is involved in the formation of polar membrane lipids in many thermophilic archaea, and the vitamin-B12-independent methionine synthase (EC:2.1.1.14), functionally replacing the vitamin-B12-dependent methionine synthase (EC:2.1.1.13), which is absent from every member of this order. The DNA polymerase D (EC:2.7.7.7) was also lost in NC LCA. Both the presence of vitamin-B12-independent methionine synthase and the absence of DNA polymerase D were previously reported in *Ca*. N. islandicus 3F^42^ and *Ca*. N. cavascurensis SCU2^43^, but present results expand those findings to all members of the order. Other notable losses in this transition are the Kdp, high-affinity ATP-driven K^+^-transport system (EC:3.6.3.12), which is involved in osmotic stress resistance in low potassium environments and Uvr excinuclease, which is involved in DNA repair from ultraviolet DNA damage. Kpd is absent from almost all marine Thaumarchaeota in this analysis, indicating that this system is not essential in this environment. It has been proposed that the absence of Uvr in deep-water Nitrosopumilales is due to the lack of light in this environment^12^. This theory agrees with the absence of these genes in Nitrosocaldales, which derive from hot spring (in the case of 3F, SCU2 and J079) and deep ocean (in the case of SAT137 and UBA231) environments in which light is also likely limited.

During the transition from AOA LCA to the last common ancestor of Nitrososphaerales (LCA NS), many genes were acquired including the mismatch repair genes DNA adenine methylase (EC:2.1.1.72) and DNA helicase II (EC:3.6.4.12), possibly conferring additional resistance to DNA damage as these organisms expanded into environments with more light exposure such as surface soil and sediment. This transition also involved the loss of the *Phn* phosphonate transport system, which provides a source of phosphate in environments where alternative phosphorus sources are scarce^44^, which may be required less in more nutrient-rich soil and sediments (Supplementary Data 11 and 12).

The evolution of AOA LCA to the last common ancestor of Nitrosopumilales (NP LCA) involved gain of ubiquinone-dependent (EC:7.1.1.2) and ubiquinone-independent (EC:1.6.99.3) NADH dehydrogenases (Supplementary Data 11 and 12), giving NP LCA all three families of respiratory NADH dehydrogenases, which regenerate NAD+ from NADH by catalysing the transfer of electrons from NADH to coenzyme Q10 in oxidative phosphorylation. The advantage of encoding all three families remains largely unknown^45–47^, but could help maintain respiratory function under changing metabolic conditions.

## Discussion

As for many prokaryotic lineages, the metabolic diversity of Thaumarchaeota remains to be fully characterized, especially in underexplored environments. The 12 genomes presented in this work offer the first genome representatives for three of the six family-level clades of Nitrososphaerales. This enabled a resolution of Nitrososphaerales phylogeny that was not possible from *amo*A gene-based phylogenies and provided insights into their predicted physiology. Although most of the presently available thaumarchaeotal genomes belong to the marine groups, the majority of thaumarchaeotal phylogenetic diversity is present in non-ammonia-oxidising Thaumarchaeota and in AOA from terrestrial environments. In addition to phylogenetic diversity, substantial genome novelty is also predicted in these organisms, with the Nitrososphaerales possessing the most novel gene families in the ammonia-oxidising Thaumarchaeota, resulting from two large lateral gene transfer events in their history. The class Nitrososphaeria represent the most environmentally widespread archaea on the planet, despite their apparent specific metabolic niche for ammonia oxidation. The present work confirmed that core metabolic capacities are maintained for ammonia oxidation and autotrophy but, importantly, revealed that their diversification is linked to acquisition of many different traits to survive in diverse environments, explaining the previously suggested vast protein family differences required for environment-specific adaptation^17,29,39,43^. The transition of Thaumarchaeota to an autotrophic ammonia-oxidising lifestyle has been proposed to result from a large lateral gene transfer event in the last common ancestor of AOA^12^. The genomes presented in this study enabled the detailed analysis of the evolutionary history of Nitrososphaerales gene families, identifying two additional LGT events associated with large gene gains and extensive molecular innovation. These findings are relevant to understand the diversification of an entire phyla and suggest that further genome sampling from this group would probably allow identification of other molecular innovations.

It has been proposed that archaeal genome evolution is driven by punctuated episodes of extensive gene acquisition, followed by lineage-specific gene loss^48^. While our analysis indicates that gene transfer contributed to the evolution of Nitrososphaerales, the predominant mode of genome expansion was gene duplication of both ancestral and originating gene families, with higher duplication rates occurring in the latter. As suggested previously^49^, the duplication of newly acquired genes by LGT may facilitate higher gene dosage of a novel metabolic function, which can enable increased production of a gene product in high demand^50^. Duplicated genes can diverge sufficiently to perform a novel function^51–53^ (neofunctionalisation) or specialise to perform the same function under different environmental conditions, such as pH^54^ or temperature^55^ (subfunctionalisation). Therefore, duplication may have a role in Nitrososphaerales adaptation, allowing the maintenance of essential functions in fluctuating and heterogeneous terrestrial environments^56^. While gene duplication has been previously reported in archaea and bacteria, its importance in comparison to LGT may have been underappreciated, at least for some lineages^49^. This unexpected importance of duplication in the evolution of Nitrososphaerales led us to reinterpret the results of a previously archaea-wide evolutionary analysis^19^, which revealed that a similar pattern of large origination events (>500 gene families) and subsequent duplications (>350 duplications) may have also occurred in other archaeal lineages, especially for the lineages with the largest known archaeal genomes (including *Haloarcula marismortui* and *Haloferax volcanii*)^19^. Taken together, these results suggest that duplication of ancestrally acquired genes may be an important mechanism of genome expansion across a number of archaeal lineages. Larger genome sizes have been reported for mesophilic soil prokaryotes (both archaea and bacteria) compared to marine relatives^57^, but it is unclear whether this is due to genome expansion in soil lineages from ancestors with smaller genomes, or from genome reduction in marine lineages. This work provides an example of a case in which soil-marine genome size differences are driven by genome expansion in soil lineages. While gene duplication is a well-documented mechanism in eukaryotic evolution^58^, demonstration of its importance in thaumarchaeotal evolution requires further investigation across other microbial lineages.

## Methods

### Sampling, sequencing and genome assembly

Sediment samples were taken from 23 sites spread across 7 tributaries of the River Thames, UK, at three different times, with duplicate samples taken in the summer of 2015 and triplicate samples taken in the summer and winter of 2016. DNA extraction was performed from 0.5 g of sample within 12 h of collection using the FastDNA^TM^ spin kit for soil, following manufacturer’s protocol. Libraries were constructed with TruSeq^®^ DNA library preparation kit and sequenced on the HiSeq2500 platform (2 × 150 bp reads). Sequences were obtained from 171 samples at an average read depth of 45 million, resulting in 2.3 Tbp. The 171 samples were co-assembled using MEGAHIT^59^ with default parameters giving a 59.3 Gbp assembly with an N50 of 999 bp and a total of 4,340,028 contigs >2000 bp in length. Reads from every sample were mapped back onto these contigs using bwa-mem^60^ with default parameters and coverage depths were calculated. These coverage depths and the sequence composition of the contigs were used by the CONCOCT clustering algorithm^61^ to produce 6008 genomic bins. Twelve of these bins were found to possess at least one of the *amo*A or *amo*B genes that are characteristic of ammonia-oxidizing archaea, detected using BLASTn^62^ against custom databases of *amo*A and *amo*B sequences^21^.

### Sequence database

Thaumarchaeotal (both AOA and non-AOA lineages) genomes were searched in literature and downloaded from NCBI (www.ncbi.nlm.nih.gov) and IMG (https://img.jgi.doe.gov/) genome reference databases (final data collection in May 2019). In addition, 12 MAGs from the Thames river were assembled in this study. Thirteen diverse genomes of the related archaeal lineages Aigarchaeota (11 genomes) and Bathyarchaeota (two genomes) were also added to this database. All genomes were annotated using Prokka v1.11^63^ to ensure uniformity between annotation methods and genome completeness and contamination were estimated using CheckM^64^. Only genomes with completeness higher than 45% and presenting <10% contamination were kept in the final dataset (to avoid bias in phylogenomic analyses), reaching a total of 152 thaumarchaeotal genomes (29 culture genomes, 51 single-cell genomes and 72 MAGs (Supplementary Data 1)). These completeness and contamination thresholds were based on a balance between genome completeness and taxonomic coverage, and were chosen specifically for this work, and may not be appropriate benchmarks for other evolutionary genomic studies. Several genome characteristics were estimated, including GC content (using QUAST^65^), total predicted genomic size (measured genome size corrected by the completeness score) and predicted optimal growth temperature (based on a machine learning model Tome^66^). Environmental sources were also retrieved from the genome reference databases or from the associated published study (Supplementary Data 1). As detailed current phylogeny of Thaumarchaeota is based on the *amo*A or 16S rRNA genes^2,21,25^, these two phylogenetically congruent markers^21^ were retrieved using RNAmmer^67^ or BLASTp^62^ against two representative *amo*A databases^2,25^. Their classification towards the most up-to-date Thaumarchaeota *amo*A and 16S rRNA gene databases^2,21^ was performed using BLASTn^62^. Initial classification of genome sequences was performed using classify_wf in the GTDB-Tk package^68^.

### Phylogenomic reconstruction and proteome content changes across evolutionary history

#### Datasets

Two datasets were used in this study: a phylum-level phylogenomic reconstruction including 165 genomes with a completeness >45% and contamination lower than 10%, and Nitrososphaerales-level phylogenomic reconstruction performed on the 26 genomes belonging to this lineage.

#### Ortholog selection

The best of three ortholog selection methodologies was selected (see SI: Extended phylogenomics). Ortholog groups (OGs) were detected using Roary (-i 50, -iv 1.5)^69^ and core OGs were defined as those that were present in a single copy in each genome and were present in at least 85% of the near complete genomes. Core OGs were aligned individually using MAFFT L-INS-i^70^ and spurious sequences and poorly aligned regions were removed with trimal (automated1, resoverlap 0.55 and seqoverlap 60)^71^. Alignments were removed from further analysis if they presented evidence of recombination using the PHItest^72^. Such ortholog prediction using the MCL algorithm resulted in 75 and 188 marker genes for the phylum-level and the Nitrososphaerales-level phylogenomic reconstruction, respectively (workflow illustrated in Supplementary Fig. 6).

#### Phylogenomic tree construction

Phylogenomic reconstruction was performed on each dataset, using a concatenated supermatrix of core OG alignments. Each maximum likelihood tree was constructed with IQ-TREE^73^ using the best-fitting protein model predicted in ModelFinder^74^ and an edge-linked partition model (model details in Supplementary Data 13). Branch supports were computed using the SH-aLRT test^75^ and 2000 UFBoot replicates and a hill-climbing nearest neighbour interchange (NNI) search was performed to reduce the risk of overestimating branch supports. Trees were rooted with the Bathyarchaeota genomes.

#### Proteome content changes across evolutionary history

For the phylum-level dataset, protein families were detected with Roary with reduced stringency (-i 35, –iv 1.3, –s) and sequences shorter than 30 amino acids and families with <4 sequences were removed from further analysis. All remaining sequences within each family were aligned using MAFFT L-INS-I, processed with trimal (automated1) and ML phylogenetic trees were constructed for each alignment (using IQ-TREE^73^ with 1000 UFBoot replicates, a NNI search and the best substitution model selected by ModelFinder^74^). Ninety-seven percent (5683 of the 5850) of the protein family trees could be probabilistically reconciled against the supermatrix tree using the ALEml_undated algorithm of the ALE package^20^ to infer the numbers of duplications, losses, intra-LGT (transfer within the sampled genome set) and originations (including both inter-LGT, e.g. transfer from other phyla outside the sampled genome set or de novo gene formation) on each branch of the supermatrix tree. Genome incompleteness was probabilistically accounted for within ALE using the genome completeness values indicated by CheckM^64^. For the purposes of this analysis the small number of genes transferred between the related phyla Thaumarchaeota, Aigarchaeota and Bathyarchaeota genomes studied here are deemed intra-LGT events, not origination events. Ancestral proteome content (both size and predicted metabolic pathways) was estimated for each node of the tree. All phylogenomic trees were visualised using FigTree (http://tree.bio.ed.ac.uk/software/figtree/) and iTOL^76^.

### Taxonomic affiliation

Internally consistent taxonomic levels were calculated using an agglomerative clustering approach. Average amino acid identities (AAIs) between pairwise sets of genomes were calculated using CompareM (https://github.com/dparks1134/CompareM) and AAIs hierarchical clustering was performed in MeV (https://sourceforge.net/projects/mev-tm4/) using Euclidean distance with complete linkage. Maximum divergence is defined as the lowest AAI between any two members of the same group, subtracted from 100%. Pairwise relative phylogenetic distances between each genome were subjected to hierarchical clustering using single linkage Pearson correlation and taxonomic levels corresponding to class, order and family were defined as Pearson’s distances of less than 0.34, 0.13 and 0.015, respectively. These cut-off distances were chosen by comparison to existing thaumarchaeotal taxonomy^5–8,42,43,77–81^. The taxonomic levels for genus and species levels were defined with AAI thresholds higher than 70% and 95%, respectively^82,83^. The nomenclature of the different taxonomic stratifications relied primarily on previously existing classifications, with attribution of the names being primarily based on the strains whose cultivation was first reported. For clusters with no reported culture, the family-level nomenclature inferred from previously ammonia monooxygenase phylogenetic tree^2^ was used. For groups not matching those affiliations, such as genomes lacking the ammonia monooxygenase, the first listed genome of the group was used to name the group. In addition, the topology of a subset of the phylogenomic tree (focusing on ammonia-oxidising Thaumarchaeota) was compared to previously published *amo*A-based phylogenies^2,14^ at the family level.

### Functional annotation and metabolic reconstruction

For each protein family, a medoid sequence (the sequence with the shortest summed genetic distances to all other sequences in the family) was calculated under the BLOSUM62 substitution matrix using DistanceCalculator in Phylo (https://biopython.org/wiki/Phylo). Medoids were annotated against the KEGG database^84^ using GhostKOALA^85^, against the arCOG database^86^ using Diamond BLASTp^87^ (best-hit and removing matches with e-value >10^−5^, % ID < 35, alignment length <80 or bit score <100) and against the Pfam database^88^ using hmmsearch^89^ (-T 80). Carbohydrate active enzymes were annotated using HMM models from dbCAN (http://bcb.unl.edu/dbCAN2/) (filtered with hmmscan-parser.sh and by removing matches with mean posterior probability <0.7). The predicted protein families present within each ancestor genome was inferred from the ALE results, allowing for ancestral metabolic reconstruction at each node of the phylogenetic tree. Proteome novelty in extant genomes was defined as the percentage of proteins encoded by a genome that do not have a homologue in the arCOG database. Gene family originations were predicted to be the result of inter-LGT if the medoid of the family possesses homologues in a database of UniRef90^90^ sequences with strain-level designations and excluding thaumarchaeotal matches. The origins of these putatively laterally acquired gene families were estimated using the best match in this database.

### Reporting summary

Further information on research design is available in the Nature Research Reporting Summary linked to this article.

## Supplementary information

Supplementary Information

Peer Review File

Reporting Summary

Description of Additional Supplementary Files

Supplementary Data 1-16

## Data Availability

Genome sequences assembled in this work are available from Genbank under the accession numbers JAATVI000000000 (Nitrososphaerales archaeon TH1173), JAATVJ000000000 (Nitrososphaerales archaeon TH1177), JAATVK000000000 (Nitrososphaerales archaeon TH5894), JAATVL000000000 (Nitrososphaerales archaeon TH703), JAATVM000000000 (Nitrososphaerales archaeon TH1920), JAATVN000000000 (Nitrososphaerales archaeon TH5888), JAATVO000000000 (Nitrososphaerales archaeon TH4187), JAATVP000000000 (Nitrososphaerales archaeon TH5896), JAATVQ000000000 (Nitrososphaerales archaeon TH1917), JAATVR000000000 (Nitrososphaerales archaeon TH526), JAATVS000000000 (Nitrososphaerales archaeon TH5895) and JAATVT000000000 (Nitrososphaerales archaeon TH5893). Public data is available from NCBI (www.ncbi.nlm.nih.gov), IMG (https://img.jgi.doe.gov/), KEGG (https://www.genome.jp/kegg/), dbCAN (http://bcb.unl.edu/dbCAN2/download/) and arCOG (https://ftp.ncbi.nih.gov/pub/wolf/COGs/arCOG/).

## Code availability

Custom scripts for manipulating ALE outputs have been deposited on GitHub at https://github.com/Tancata/phylo/tree/master/ALE (10.5281/zenodo.4012549)^91^.
