# Supplementary material for: Gene duplication drives genome expansion in a major lineage of Thaumarchaeota
Source: Nat Commun. 2020 Oct 30;11:5494. doi: 10.1038/s41467-020-19132-x (PMC7603488; doi:10.1038/s41467-020-19132-x)
Supplement: Supplementary file 1 — Supplementary Information [file 41467_2020_19132_MOESM1_ESM.pdf]

Supplementary Information: Gene duplication drives genome expansion in a major lineage of Thaumarchaeota

Paul O. Sheridan[1,2], Sebastien Raguideau[3], Christopher Quince[3], Jennifer Holden[4], Lihong Zhang[5], Thames Consortium[*], Tom A. Williams[2], Cécile Gubry-Rangin[1,#]

Supplementary Methods

Extended Phylogenomics

Estimating phylogeny: In order to establish a robust phylogeny of the Thaumarchaeota, three methods of phylogenetic marker gene selection were used. In the first method, 43 marker genes were selected using CheckM[1]. One ortholog group was removed, as it presented evidence of recombination using the PHItest[2] and the remaining 42 genes were used as marker genes. In the second method, the Hidden Markov Model (HMM) profiles of the 122 archaeal phylogenetic markers described by GTDB[3] were downloaded from Pfam[4] and TIGRFAMS[5] and searched for in the genomes using hmmsearch[6] (-T 80). For the 80 matching proteins that were present in single copy in at least 70% of the genomes, the regions aligning to the HMM profiles were extracted and used as marker genes. In the third method, orthologs were detected using the MCL algorithm-based software, Roary[7] (-i 50, -iv 1.5) and core ortholog groups were defined as those that were present in only one copy in each genome and were present in at least 85 % of the genomes presenting more than 80 % completeness and less than 5 % contamination (workflow illustrated in Supplementary Figure 6). No evidence of recombination in these orthologs groups was detected using the PHItest, resulting in 75 marker genes.

For all three datasets, marker genes were aligned individually using MAFFT L-INS-i[8] and spurious sequences and poorly aligned regions were removed with trimal[9] (automated 1, resoverlap 0.55 and seqoverlap 60). A maximum likelihood tree was constructed for a

concatenated supermatrix of alignments with IQ-TREE[10] using the best fitting protein model in ModelFinder[11] for each alignment and an edge-linked partition model. Branch validation involved 1000 SH-aLRT test[12] and 2000 UFBoot replicates, further optimised with a hill-climbing nearest neighbor interchange (NNI) search.

Comparing phylogeny estimations: Likelihoods for each of the phylogenies were calculated by amalgamated likelihood estimation of 5,683 thaumarcheotal gene families and used to perform an approximate unbiased tested of the three trees in the manner described by Williams *et al* 2017[13] and implemented in ALE[14] and CONSEL[15]. The most likely tree was used for further analysis.

Topology testing in constraint trees: Topology tests were performed with IQ-TREE using the MCL markers approach. The aim was to compare an unconstrained MCL tree with three constrained trees that represent the major incongruences between this work and the phylogenomic tree proposed by Ren *et al.* [16]: a) the constrained tree 1 (Monophyletic yellow, using the colour scheme from Supplementary Figure 7) forces FN1, YP1-bin3 and UBA183 to form a monophyletic clade with AD-613-B23, RBG-16-49-6, SAT139, EAC691, UBA57, UBA223 and SP3992; b) the constrained tree 2 (Paraphyletic blue) forces 3F and SCU2 to form a paraphyletic clade with SAT137 and UBA213; c) the constrained tree 3 (Monophyletic yellow and paraphyletic blue) possesses the constraints of both constrained tree 1 and 2.

Supplementary Results

A robust phylogeny for the Thaumarchaeota

A phylogenomic approach was adopted to explore the relationships of 152 Thaumarchaeota genomes, 11 Aigarchaeota and 2 Bathyarchaeota genomes. As the use of different phylogenetic markers and tree construction methods has been known to produce dramatically different

estimates of phylogeny, we examined three different phylogenomic marker sets and compared the inferred topologies.

The tree constructed using CheckM markers is highly similar to the phylogeny previously proposed by Ren *et al* [16]. The HMM and MCL are largely congruent with these trees, albeit with some differences. In the CheckM tree, SAT137 and UBA213 form a separate clade to J079, 3F and SCU2, whereas these organisms for a single clade in the HMM and MCL trees (Supplementary Figure 7, blue dots). In the HMM and MCL trees, FN1, YP1-bin3 and UBA183 form a separate clade to AD-613-B23, RBG-16-49-6, SAT139, EAC691, UBA57, UBA223 and SP3992, whereas using the CheckM markers these organisms form a single clade (Supplementary Figure 7, yellow dots). In the MCL tree, BS3, BS4, ARK01, UBA164, DS1 and UBA160 form a strongly supported monophyletic group. This group is also present in the HMM tree (albeit with poor support) and is split into two poorly supported groups in the CheckM tree (Supplementary Figure 7, red dots). The three trees gave incongruent topologies for the Nitrososphaerales (Supplementary Figure 7, green dots), so we performed a targeted analysis of this order using more phylogenetic markers, as discussed in the main text. The resulting topology was consistent with that obtained using the MCL markers in the phylum-wide analysis.

As the three trees are providing strong support (SH-aLRT and UFBoot) for some contradicting phylogenies, an additional tree was performed. Different species tree estimations imply different scenarios of gene family evolution, resulting in different gene family likelihoods when using a probabilistic gene tree to species tree reconciliation model. It was therefore possible to use an approximately unbiased test to establish a confidence values for the three alternative phylogeny estimations. From this analysis, it was possible to reject all but the MCL tree (Supplementary Data 14).

Additionally, topology tests were performed using the MCL markers that compared the unconstrained MCL tree to three constrained trees that represented two of the incongruent placements observed between the CheckM tree and the other two trees, namely splitting of the blue dotted clade and merging of the yellow dotted clades from Supplementary Figure 7. The combination of monophyletic yellow clade and paraphyletic blue clade could be significantly rejected by approximate unbiased testing and several other tests (Supplementary Data 15). While the individual incongruences could not be statistically rejected by this method, they were far less favoured than the unconstrained MCL tree topology by all statistical tests applied here (Supplementary Data 15).

Finally, as part of the phylogenomic workflow described in Supplementary Figure 6, a species tree was formed using the same marker genes but including only the 87 genomes sequences of greater than 80 % completeness and less than 5 % contamination. The resulting tree is largely in agreement with the full 165 genome species tree, with no strongly supported contradictions (Supplementary Figure 8). This indicates that missing data in the 165 genome supermatrix, resulting from some genomes lacking some of the markers, has not had a major effect on the topology of the species tree.

Extended optimal growth temperature

In silico prediction of optimal growth temperature (OGT) was performed with Tome, a machine-learning model that uses 2-mer amino acid composition across an organism proteome. Results obtained from this analysis were validated against a set of Thaumarchaeota for which the OGT has been experimentally determined. Tome proved to be relatively accurate in predicting OGT for the mesophilic (defined with an OGT range of 20-40 °C) Thaumarchaeota, with an average difference of 5 °C between the experimental and *in silico* estimations

(Supplementary Data 16). However, this difference increases to an average of 10 °C in the thermophiles (defined with an OGT range of 41-122 °C) (Supplementary Data 16), even if predicted values were related to experimental values (R^2=0.88). Therefore, Tome can reasonably predict the mesophilic or thermophilic state of an organism from the genome, but OGT may be significantly higher than predicted in thermophiles.

Effect of MAG contamination on gene duplication estimates

One concern when extensively using MAGs in an analysis is that genomes may be contaminated by closely related strains, due to their similar DNA composition, which in this analysis could potentially result in artificially inflated gene duplication values. However, this is unlikely to have occur in this work for three main reasons. Firstly, the greatest duplication events (indicated by nodes A, B, C and D in Figure 3) occurred in non-terminal branches, meaning strain heterogeneity would need to have occurred in a similar manner, in the same genes, in multiple MAGs to account for the duplication event values. This is very unlikely, as exemplified in the second largest duplication event (node D in Figure 3), as this phylogenetic clade contains a single MAG and three nearly complete genomes reconstructed from thaumarchaeotal culture (each with 0% strain heterogeneity). Secondly, the percentage of gene families that underwent duplication is similar in all genomes within this clade (both MAG and 3 culture-based genomes) (Supplementary Data 9). Thirdly, the binning of these MAGs was not based on DNA composition alone, but also availed of coverage information from 171 metagenome sequences. This coverage information was further investigated in TH1173 and TH5895 (Supplementary Figure 9), which are MAG descendants of the two largest duplication hotspots (nodes B and D in Figure 3). Non-metric multi-dimensional scaling of the coverage profiles show that the majority of duplicated genes (67% for TH1173 and 80% for TH5895)

are within the 50% quantile of all genes (Supplementary Figure 9), further indicating that incorrect binning together of closely related organisms is unlikely to be the cause of the high levels of duplication seen in these organisms.

Supplementary Figures.

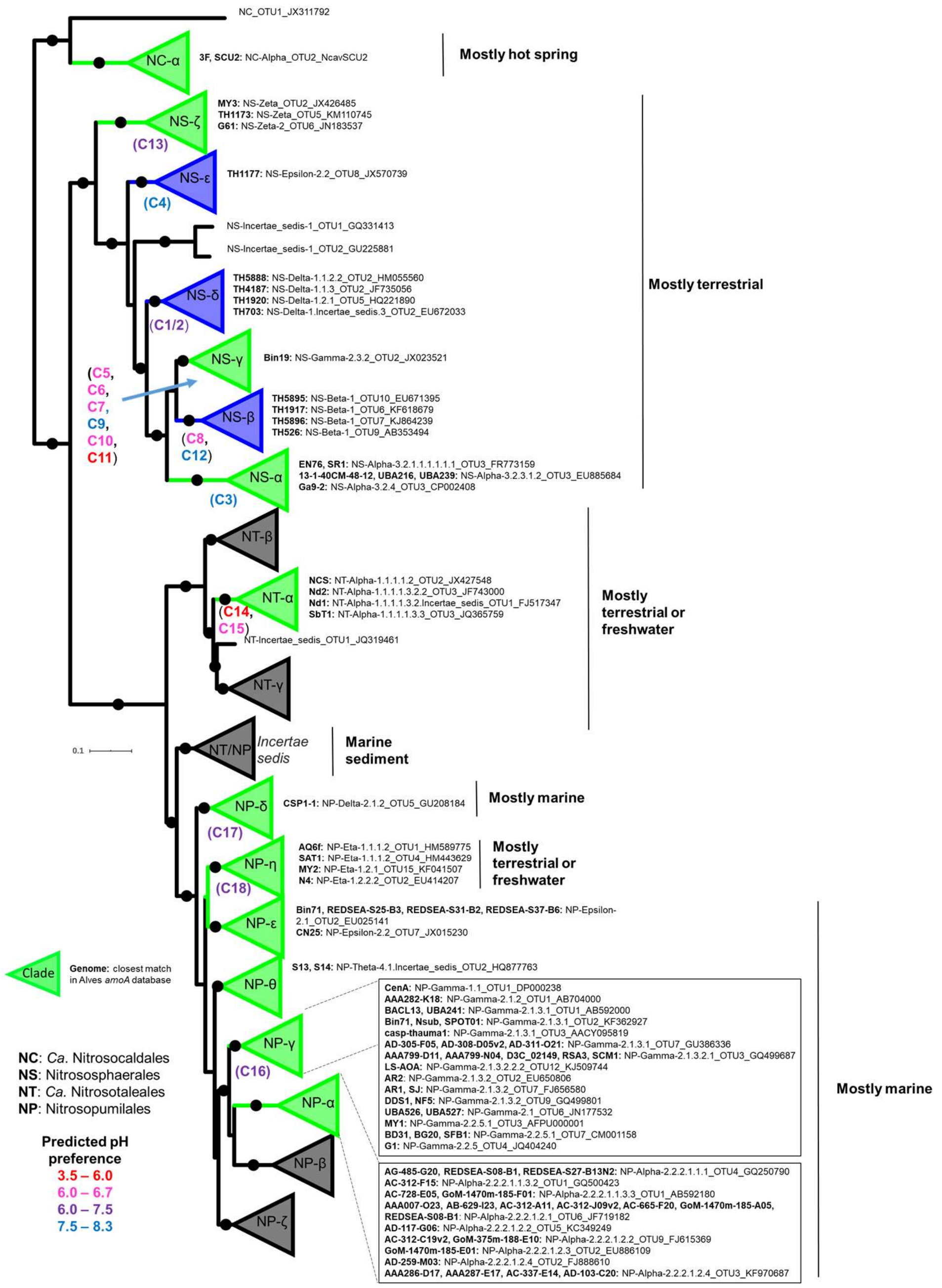

Supplementary Figure 1. Ammonia-oxidising archaea genomes in relation to the most up-to-date *amoA*-based phylogeny. This tree was reconstructed from the Newick tree file published in Alves *et al* 2018[17] and major clades that are first represented by new genomes in this work are in blue. Clades which are represented by previously publically available genomes are in green. Clades not represented by genomes in this dataset are in grey. Genomes with an *amoA* gene with at least 96% BLASTn sequence similarity to the Alves database are presented with the closest matching OTU. Dots on branches represent UFBoot support of at least 95%. The scale bar represents 0.1 substitutions per nucleotide position. The distribution of *amoA* genes from Alves database in different environments is indicated to the right of the clade. Gubry-Rangin *et al* 2015[18] *amoA* classifications from soil are in brackets under each clade based on a BLASTn approach towards the Alves database and the pH niche specialisation of each clade as predicted in Vico Oton *et al* 2016[19] is indicated by font colour.

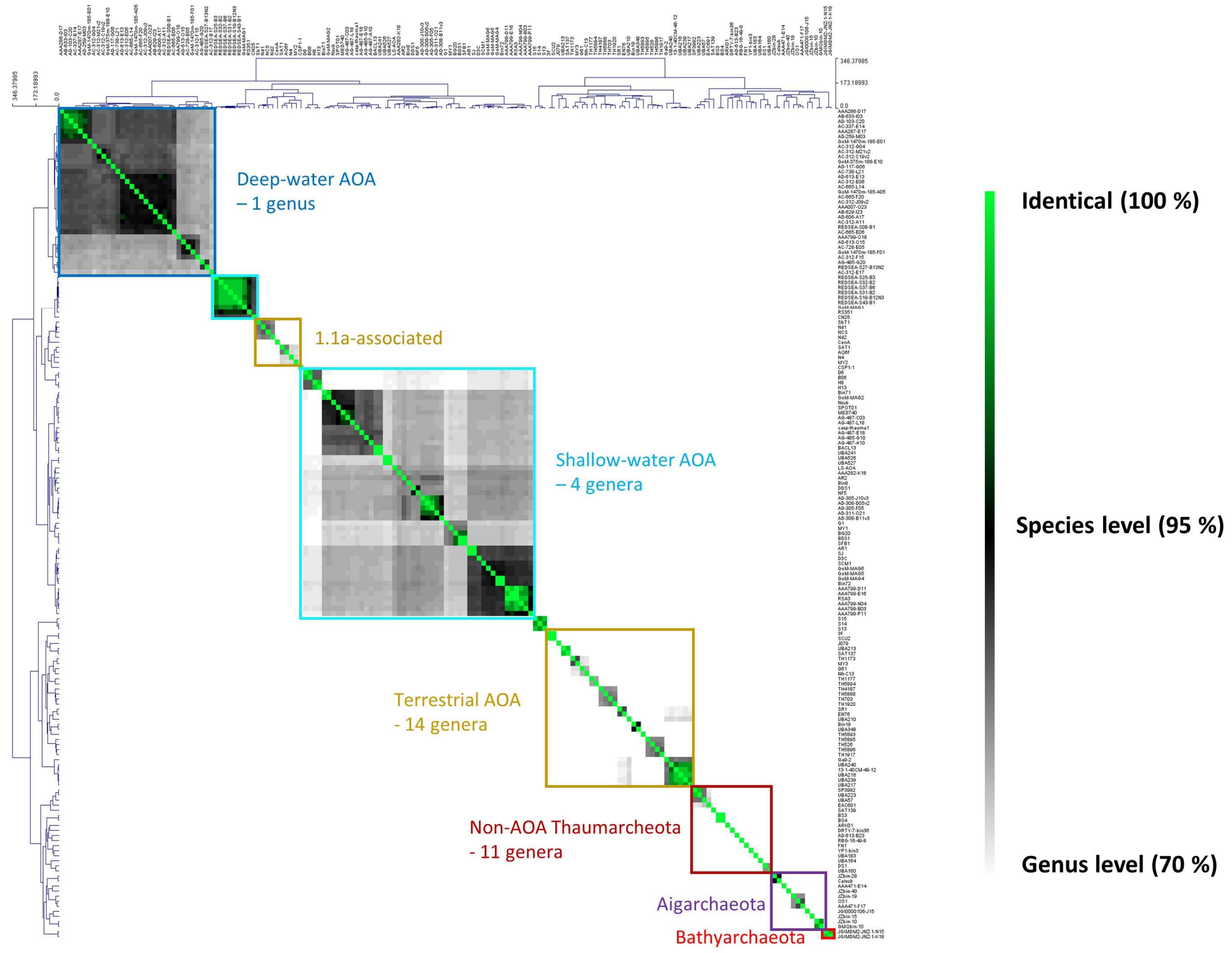

Supplementary Figure 2. Average amino acid identity of Thaumarchaeota and related archaea. Average amino acid identity (AAI) was used as a measure of genome similarity. Pairwise AAIs were clustered using Euclidean distance with complete linkage. AAIs were coloured at the species level (> 95 %) from green to black and at the genus level (> 70 %) from black to light grey. AAI values below 70% are coloured white.

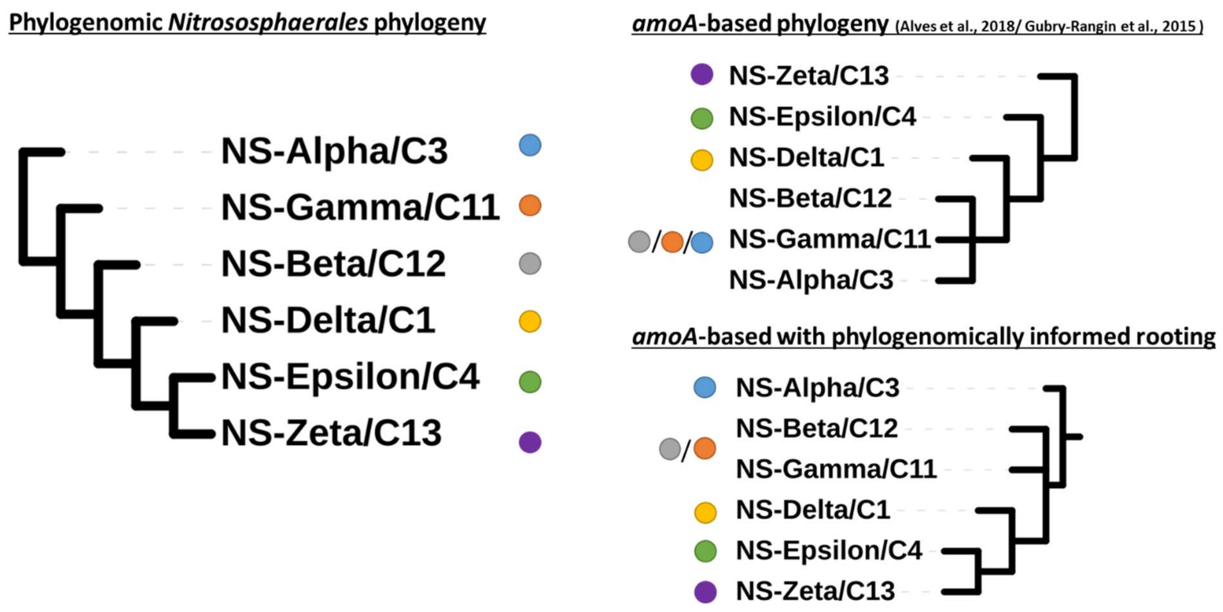

Supplementary Figure 3. Phylogenomic vs *amoA*-based phylogeny of Nitrososphaerales. For the phylogenomic and the Alves *amoA*-based phylogenies[17], branches with less than 95% UFBoot and 85% SH-aLRT were collapsed, while branches with less than 75% posterior probability were collapsed for the Gubry-Rangin *amoA*-based phylogeny[18]. Both *amoA*-based phylogenies were identical at family level. Coloured circles are used to emphasise the family-level groups. Slashes (/) indicate were branches could not be confidently resolved.

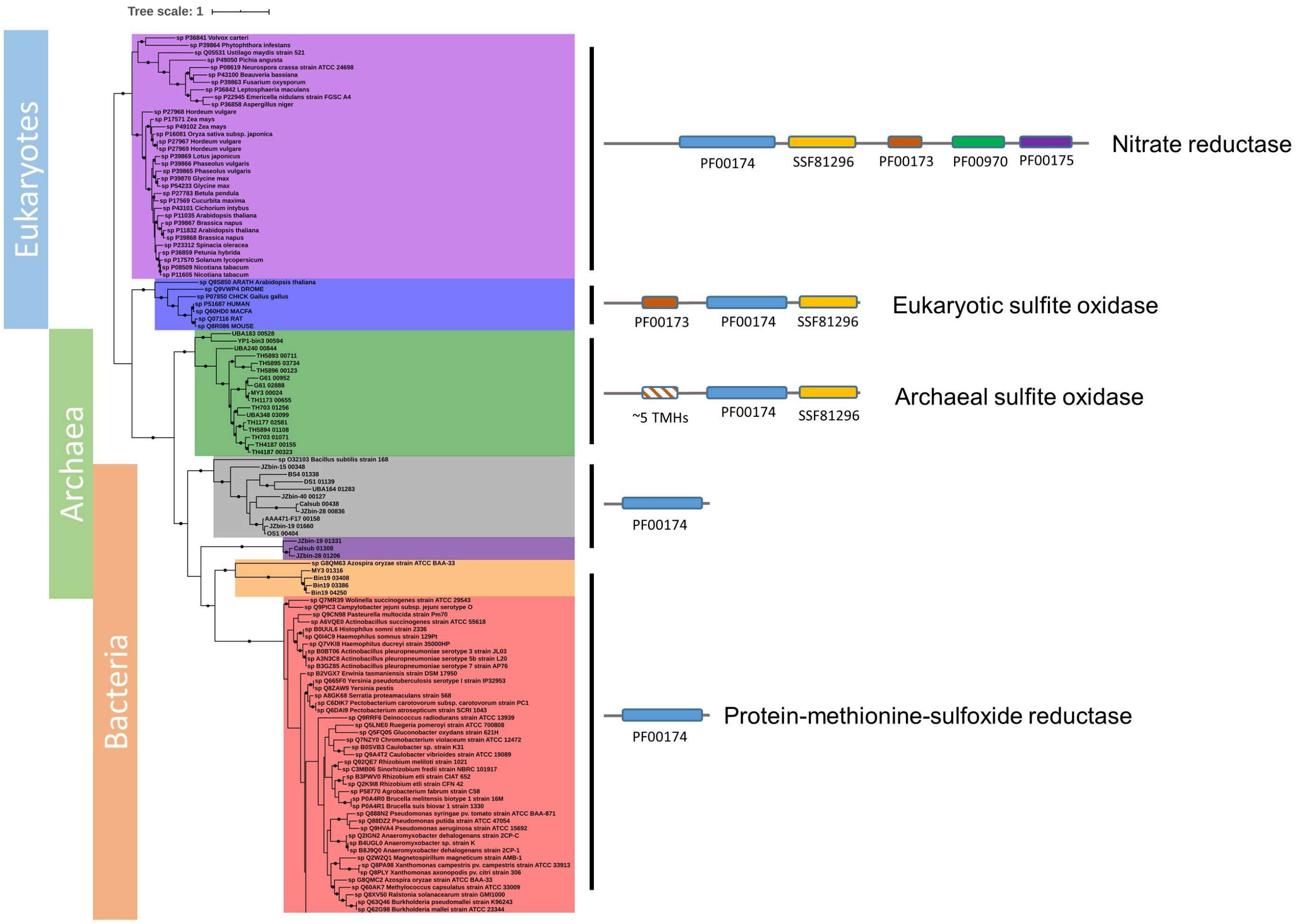

Supplementary Figure 4. Sulfite oxidase family molybdoenzymes of Thaumarchaeota. MoZymes of Thaumarchaeota represent a putative archaeal class of sulfite oxidases. Dots indicate branches with greater than 95% of 2,000 UFBoot replicates. Swiss-Prot sequences possessing the PF00174 domain were used as references. Coloured bars on left indicate the domain of origin of protein clades. Overlapping of these bars indicates clades with proteins from more than one domain of life. Transmembrane helices (TMHs). Figures to the right of tree are schematic representations of domain organisation in corresponding protein clade.

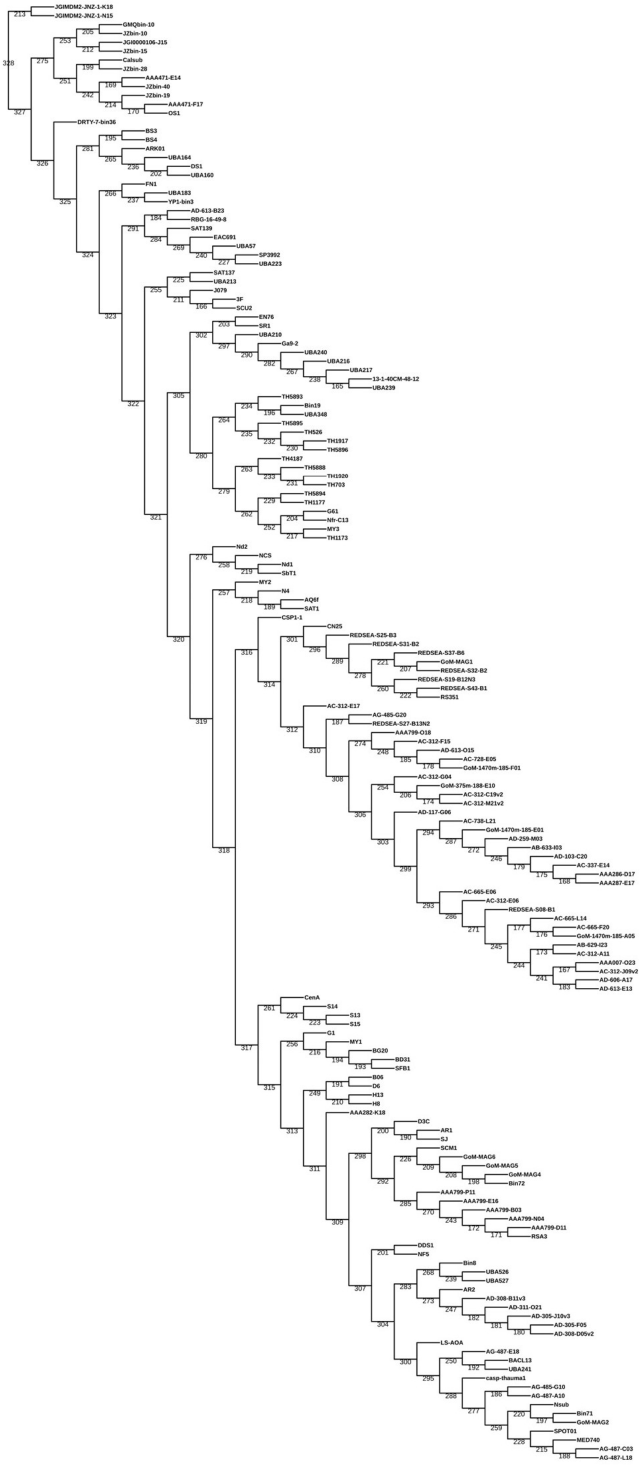

Supplementary Figure 5. Branch labelled phylogeny of Thaumarchaeota and related species. This cladogram possesses the topology of the tree presented in Figure 1. The tree branch numbers corresponded to the quantification of gene content change mechanisms in Supplementary Data 4. These two datasets were combined to form Figure 3.

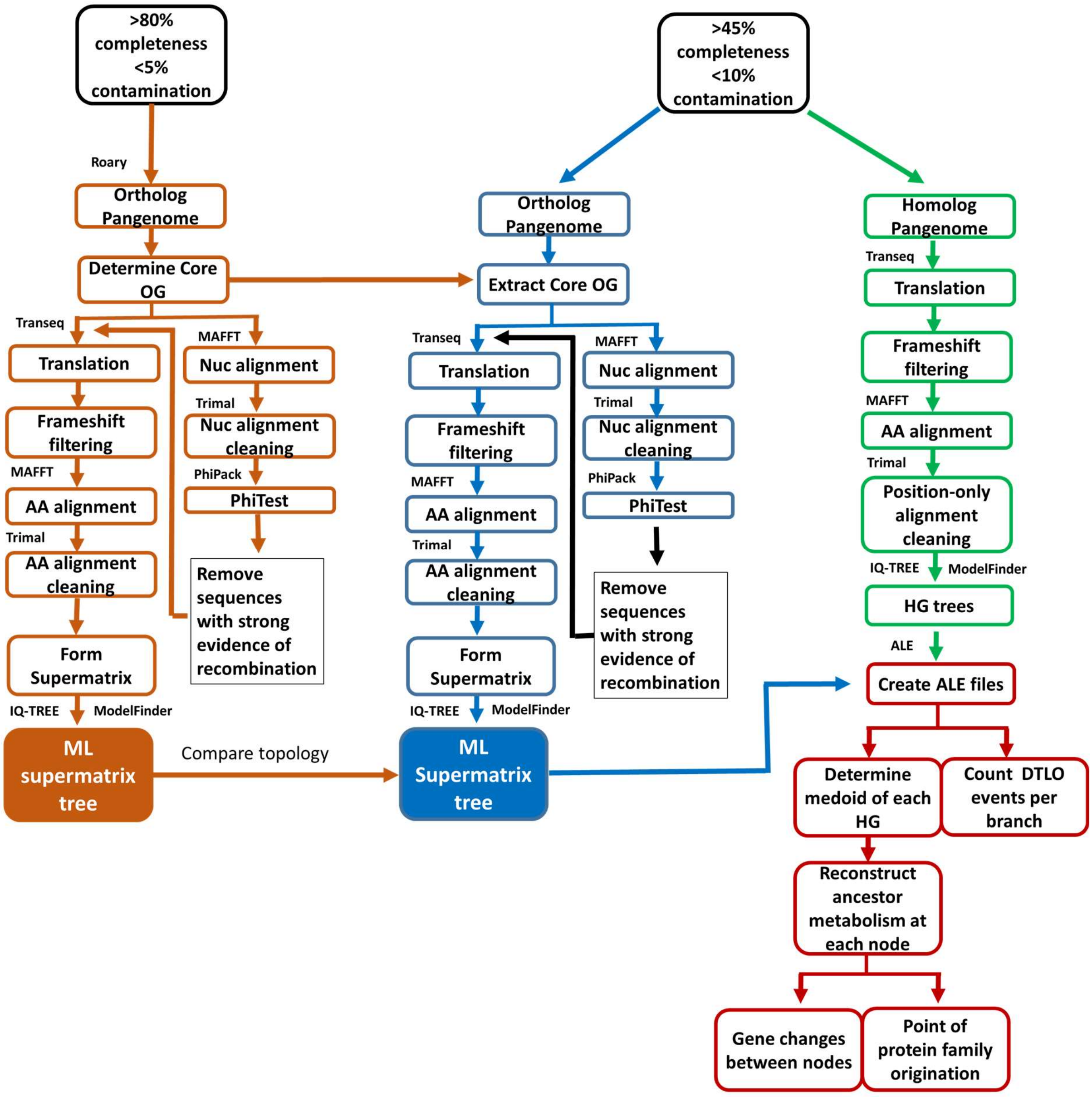

Supplementary Figure 6. Schematic workflow of phylogenomic and evolutionary analysis. This workflow describes the steps taken in the construction of the ML tree presented in Figure 1 and in gene family history prediction used to construct the Figure 2. Tools used in the orange, blue and green branches of the workflow have been shown next to their stage of use. Custom scripts for manipulating ALE outputs have be deposited at https://github.com/Tancata/phylo/tree/master/ALE .

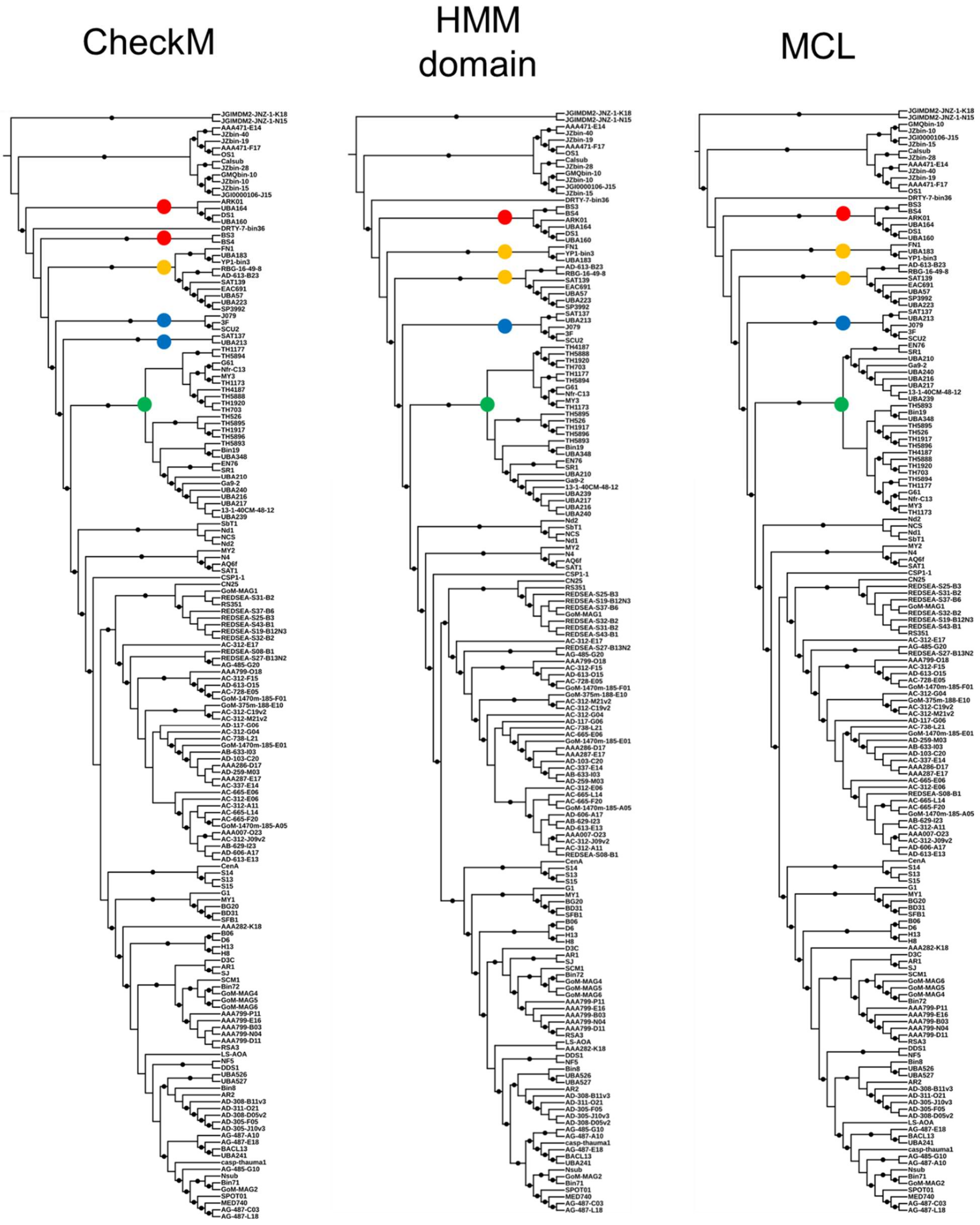

Supplementary Figure 7. ML trees using 3 methods of phylomarker selection. Coloured circles are used to highlight areas of incongruence between methods. Dots indicate branches with greater than 95% of 2,000 UFBoot and 1,000 SH-aLRT replicates. The tree is rooted with the Bathyarchaeota strains, JGIMDM2-JNZ-1-K18 and JGIMDM2-JNZ-1-N15.

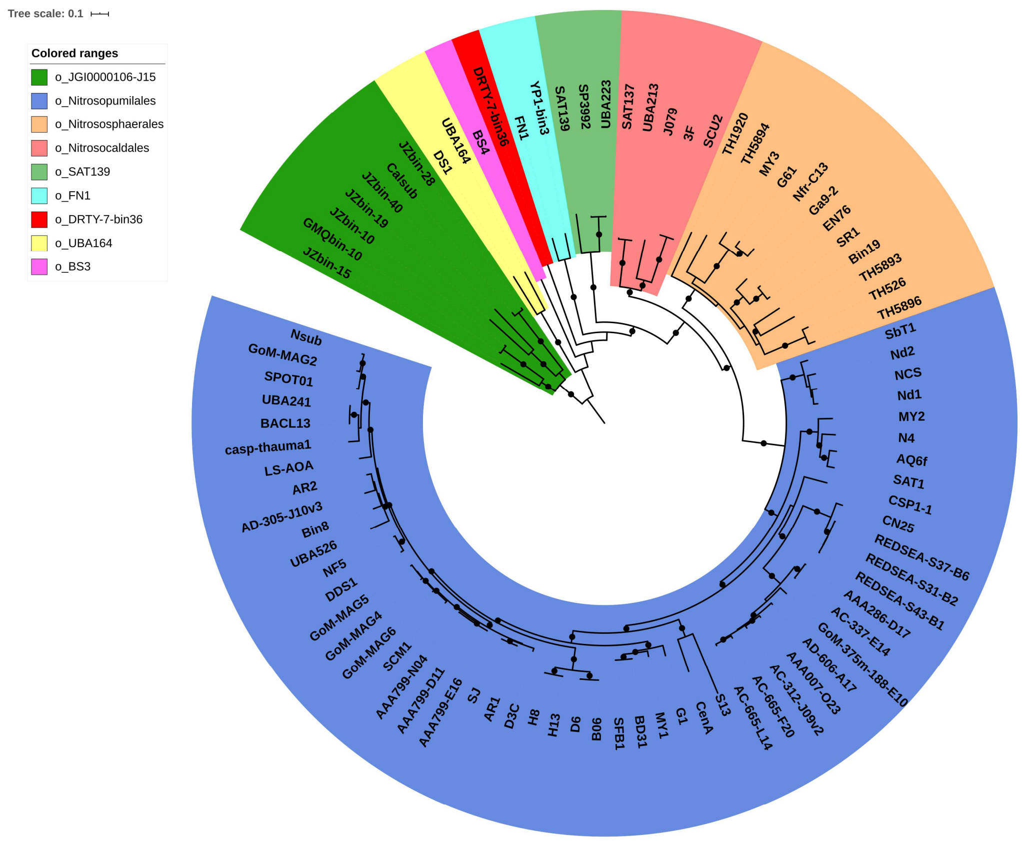

Supplementary Figure 8. Phylogeny of near complete Thaumarchaeotal and related archaeal genomes. ML tree of 75 concatenated ortholog groups. Dots indicate branches with greater than 95% of 2,000 UFBoot and 1,000 SH-aLRT replicates. Colours indicate order-level groups. The tree is rooted with the Aigarchaeota strains (o_JGI0000106-J15).

A. TH1173 B. TH5895

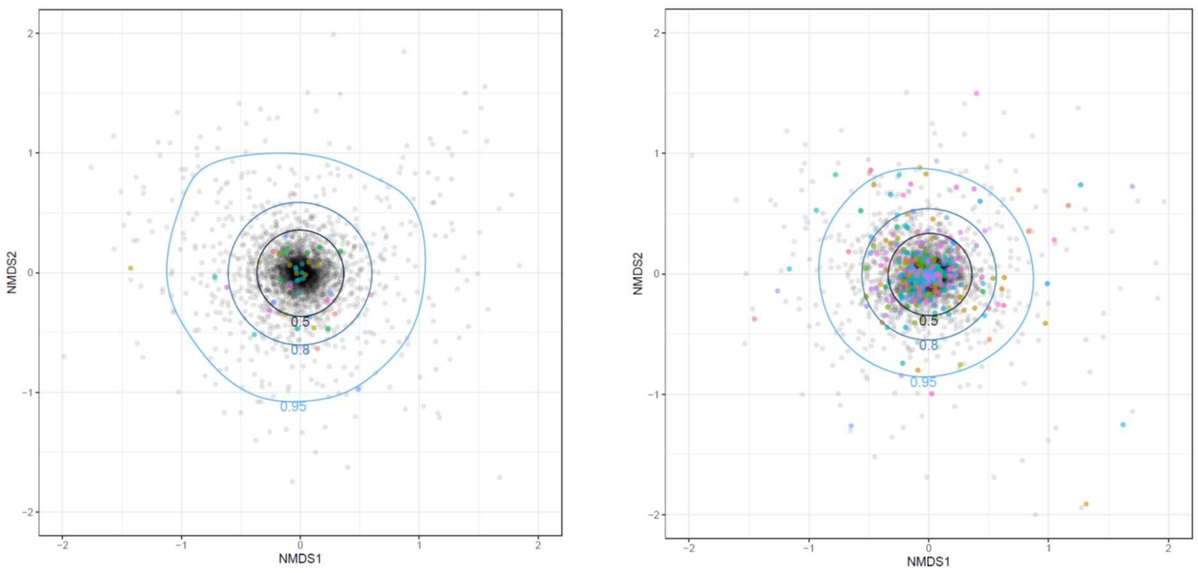

Supplementary Figure 9. Coverage profile non-metric multi-dimensional scaling of all genes from MAGs (A) TH1173 and (B) TH5895 in 171 metagenome sequences. Each group of predicted duplicated genes are represented by the same colour and non-duplicated genes are represented in grey. Contours of 0.5, 0.8 and 0.95 correspond quantiles of 50%, 80% and 95% of all genes in the corresponding MAG. Plots where produced with vegan[20] (2.5.5) and ggplot2[21] (3.3.2) within R[22] (3.4.4). Quantile contours where obtained using two-dimensional kernel density estimation from MASS[23] (7.3.51.4), taking 400 grid points and a bandwidth of 1.

Supplementary References

1. Parks, D. H., Imelfort, M., Skennerton, C. T., Hugenholtz, P. & Tyson, G. W. CheckM: assessing the quality of microbial genomes recovered from isolates, single cells, and metagenomes. *Genome Res.* **25**, 1043-1055 (2015).

2. Bruen, T. & Bruen, T. PhiPack: PHI test and other tests of recombination. *McGill University, Montreal, Quebec* (2005).

3. Parks, D. H. *et al.* A standardized bacterial taxonomy based on genome phylogeny substantially revises the tree of life. *Nat. Biotechnol.* **36**, 996-1004 (2018).

4. El-Gebali, S. *et al.* The Pfam protein families database in 2019. *Nucleic Acids Res.* **47**, D427-D432 (2019).

5. Haft, D. H. *et al.* TIGRFAMs and genome properties in 2013. *Nucleic Acids Res.* **41**, D387-D395 (2012).

6. Eddy, S. R. Profile hidden Markov models. *Bioinformatics* **14**, 755-763 (1998).

7. Page, A. J. *et al.* Roary: rapid large-scale prokaryote pan genome analysis. *Bioinformatics* **31**, 3691-3693 (2015).

8. Katoh, K. & Toh, H. Recent developments in the MAFFT multiple sequence alignment program. *Briefings in bioinformatics* **9**, 286-298 (2008).

9. Capella-Gutiérrez, S., Silla-Martínez, J. M. & Gabaldón, T. trimAl: a tool for automated alignment trimming in large-scale phylogenetic analyses. *Bioinformatics* **25**, 1972-1973 (2009).

10. Nguyen, L., Schmidt, H. A., Von Haeseler, A. & Minh, B. Q. IQ-TREE: a fast and effective stochastic algorithm for estimating maximum-likelihood phylogenies. *Mol. Biol. Evol.* **32**, 268-274 (2015).

11. Kalyaanamoorthy, S., Minh, B. Q., Wong, T. K., von Haeseler, A. & Jermiin, L. S. ModelFinder: fast model selection for accurate phylogenetic estimates. *Nature methods* **14**, 587 (2017).

12. Guindon, S. *et al.* New algorithms and methods to estimate maximum-likelihood phylogenies: assessing the performance of PhyML 3.0. *Syst. Biol.* **59**, 307-321 (2010).

13. Williams, T. A. *et al.* Integrative modeling of gene and genome evolution roots the archaeal tree of life. *Proc. Natl. Acad. Sci. U. S. A.* **114**, E4602-E4611 (2017).

14. Szöllősi, G. J., Rosikiewicz, W., Boussau, B., Tannier, E. & Daubin, V. Efficient exploration of the space of reconciled gene trees. *Syst. Biol.* **62**, 901-912 (2013).

15. Shimodaira, H. & Hasegawa, M. CONSEL: for assessing the confidence of phylogenetic tree selection. *Bioinformatics* **17**, 1246-1247 (2001).

16. Ren, M. *et al*. Phylogenomics suggests oxygen availability as a driving force in Thaumarchaeota evolution. *The ISME journal* **13**, 2150-2161 (2019).

17. Alves, R. J. E., Minh, B. Q., Urich, T., von Haeseler, A. & Schleper, C. Unifying the global phylogeny and environmental distribution of ammonia-oxidising archaea based on amoA genes. *Nature communications* **9**, 1-17 (2018).

18. Gubry-Rangin, C. *et al*. Coupling of diversification and pH adaptation during the evolution of terrestrial Thaumarchaeota. *Proc. Natl. Acad. Sci. U. S. A.* **112**, 9370-9375 (2015).

19. Oton, E. V., Quince, C., Nicol, G. W., Prosser, J. I. & Gubry-Rangin, C. Phylogenetic congruence and ecological coherence in terrestrial Thaumarchaeota. *The ISME journal* **10**, 85-96 (2016).

20. Dixon, P. VEGAN, a package of R functions for community ecology. *Journal of Vegetation Science* **14**, 927-930 (2003).

21. Wickham, H. in ggplot2: elegant graphics for data analysis (Springer, 2016).

22. Team, R. C. R: A language and environment for statistical computing. (2013).

23. Venables, W. & Ripley, B. Modern applied statistics with S Springer Verlag. *New York* (2002).
