# Supplementary material for: Gene duplication drives genome expansion in a major lineage of Thaumarchaeota
Source: Nat Commun. 2020 Oct 30;11:5494. doi: 10.1038/s41467-020-19132-x (PMC7603488; doi:10.1038/s41467-020-19132-x)
Supplement: Supplementary file 3 — Reporting Summary [file 41467_2020_19132_MOESM3_ESM.pdf]

nature research

Corresponding author(s): Cécile Gubry-Rangin

Last updated by author(s): Sep 3, 2020

Reporting Summary

Nature Research wishes to improve the reproducibility of the work that we publish. This form provides structure for consistency and transparency in reporting. For further information on Nature Research policies, see Authors & Referees and the Editorial Policy Checklist .

Statistics

For all statistical analyses, confirm that the following items are present in the figure legend, table legend, main text, or Methods section.

n/a	Confirmed	
☒	☐	The exact sample size (*n*) for each experimental group/condition, given as a discrete number and unit of measurement
☒	☐	A statement on whether measurements were taken from distinct samples or whether the same sample was measured repeatedly
☒	☐	The statistical test(s) used AND whether they are one- or two-sided<br>*Only common tests should be described solely by name; describe more complex techniques in the Methods section.*
☒	☐	A description of all covariates tested
☒	☐	A description of any assumptions or corrections, such as tests of normality and adjustment for multiple comparisons
☒	☐	A full description of the statistical parameters including central tendency (e.g. means) or other basic estimates (e.g. regression coefficient) AND variation (e.g. standard deviation) or associated estimates of uncertainty (e.g. confidence intervals)
☒	☐	For null hypothesis testing, the test statistic (e.g. *F*, *t*, *r*) with confidence intervals, effect sizes, degrees of freedom and *P* value noted<br>*Give P values as exact values whenever suitable.*
☒	☐	For Bayesian analysis, information on the choice of priors and Markov chain Monte Carlo settings
☒	☐	For hierarchical and complex designs, identification of the appropriate level for tests and full reporting of outcomes
☒	☐	Estimates of effect sizes (e.g. Cohen's *d*, Pearson's *r*), indicating how they were calculated

Our web collection on statistics for biologists contains articles on many of the points above.

Software and code

Policy information about availability of computer code

Data collection	No software was used for data collection.
Data analysis	Custom scripts have been deposited at https://github.com/Tancata/phylo/tree/master/ALE. Open source software used in analysis is referenced in Materials and Methods: MEGAHIT v1.1.3, bwa-mem v0.7.17, CONCOCT v0.4.1 , BLASTn v2.9.0, Prokka v1.11, CheckM v1.1.2, QUAST v5.0.2, Tome v2.0, RNAmmer v1.2, BLASTp v2.9.0, GTDB-Tk v89, Roary v3.12.0, MAFFT v7.407, Trimal v1.4.1, PHItest v1.1, IQ-TREE v1.6.11, ALE v1.0, FigTree v1.4.3, iTOL, CompareM v0.1.1, MeV v4.9.0, Phylo v0.58, GhostKOALA, HMMER v3.2.1.

For manuscripts utilizing custom algorithms or software that are central to the research but not yet described in published literature, software must be made available to editors/reviewers. We strongly encourage code deposition in a community repository (e.g. GitHub). See the Nature Research guidelines for submitting code & software for further information.

Data

Policy information about availability of data

All manuscripts must include a data availability statement. This statement should provide the following information, where applicable:

- Accession codes, unique identifiers, or web links for publicly available datasets
- A list of figures that have associated raw data
- A description of any restrictions on data availability

Genome sequences assembled in this work are available from Genbank under the accession numbers JAATVI000000000 (Nitrososphaerales archaeon TH1173), JAATVJ000000000 (Nitrososphaerales archaeon TH1177), JAATVK000000000 (Nitrososphaerales archaeon TH5894), JAATVL000000000 (Nitrososphaerales archaeon TH703), JAATVM000000000 (Nitrososphaerales archaeon TH1920), JAATVN000000000 (Nitrososphaerales archaeon TH5888), JAATVO000000000 (Nitrososphaerales archaeon TH4187), JAATVP000000000 (Nitrososphaerales archaeon TH5896), JAATVQ000000000 (Nitrososphaerales archaeon TH1917), JAATVR000000000 (Nitrososphaerales archaeon TH526), JAATVS000000000 (Nitrososphaerales archaeon TH5895) and JAATVT000000000 (Nitrososphaerales archaeon TH5893).

Field-specific reporting

Please select the one below that is the best fit for your research. If you are not sure, read the appropriate sections before making your selection.

☐ Life sciences ☐ Behavioural & social sciences ☒ Ecological, evolutionary & environmental sciences

For a reference copy of the document with all sections, see nature.com/documents/nr-reporting-summary-flat.pdf

Ecological, evolutionary & environmental sciences study design

All studies must disclose on these points even when the disclosure is negative.

Study description	Presentation and genomic analysis of novel metagenome-assembled genomes sequences from the ammonia-oxidising archaea and evolutionary analysis of the thaumarchaeotal phylum by gene tree - species tree reconciliation techniques.
Research sample	Analysis involved 152 thaumarchaeotal genome sequences (including 12 novel genomes from this study), eleven Aigarchaeota and two Bathyarchaeota. The 12 novel genome sequences were sequenced from sediments along the Thames river, UK. The publicly available genome sequences were sequenced from a wide variety of environmental sources, including marine, soil and hot spring environments. This dataset is intended to represent the Thaumarchaeota phylum with related outgroups and comprises all publicly available thaumarchaeotal genomes with genome completeness > 45% and contamination < 10% as of May 2019.
Sampling strategy	Not applicable as this was a study of genome evolution rather than a comparison of different environments.
Data collection	Public Thaumarchaeota genome sequences were downloaded from IMG (https://img.jgi.doe.gov/) and NCBI (www.ncbi.nlm.nih.gov) by Dr Paul O. Sheridan onto a local computer system.
Timing and spatial scale	Final data collection in May 2019
Data exclusions	Thaumarchaeotal genomes with a completeness lower than 45 % or contamination greater than 10 % were excluded from the study. These thresholds were chosen specifically for this dataset.
Reproducibility	Not applicable as this was a comparative genomic study
Randomization	Not applicable as this was a comparative genomic study
Blinding	Not applicable as this was a comparative genomic study

Did the study involve field work? ☐ Yes ☒ No

Reporting for specific materials, systems and methods

We require information from authors about some types of materials, experimental systems and methods used in many studies. Here, indicate whether each material, system or method listed is relevant to your study. If you are not sure if a list item applies to your research, read the appropriate section before selecting a response.

Materials & experimental systems

n/a	Involved in the study
☒	☐ Antibodies
☒	☐ Eukaryotic cell lines
☒	☐ Palaeontology
☒	☐ Animals and other organisms
☒	☐ Human research participants
☒	☐ Clinical data

Methods

n/a	Involved in the study
☒	☐ ChIP-seq
☒	☐ Flow cytometry
☒	☐ MRI-based neuroimaging
