# Supplementary material for: Gene duplication drives genome expansion in a major lineage of Thaumarchaeota
Source: Nat Commun. 2020 Oct 30;11:5494. doi: 10.1038/s41467-020-19132-x (PMC7603488; doi:10.1038/s41467-020-19132-x)
Supplement: Supplementary file 4 — Description of Additional Supplementary Files [file 41467_2020_19132_MOESM4_ESM.pdf]

Description of Additional Supplementary files

Supplementary Data 1. Genome descriptions. MAG and SAG indicate metagenome assembled genome and single-cell assembled genome, respectively. Protein novelty is defined as the percentage of encoded proteins that lack a close homolog (e-value < 10-5, % ID > 35, alignment length > 80 and bit score > 100) in the arCOG database.

Supplementary Data 2. Taxonomic stratification of Thaumarchaeota. Grey columns display the closest match of query *amo*A and 16S rRNA genes to sequences in Thaumarchaeota phylogenetic databases.

Supplementary Data 3. GTDBTk classify_wf classification. Relative evolutionary divergence was calculated using the Genome taxonomy database toolkit available at https://github.com/Ecogenomics/GTDBTk.

Supplementary Data 4. Quantified mechanisms of proteome change. Gene content changes on each branch of the phylogenomic tree presented in Figure 1 have been divided into four evolutionary mechanisms of change: duplications, losses, intra-LGT and originations. Duplication and loss are defined as the copying and loss of a gene within a genome, respectively. Intra-LGT is defined as the acquisition of a gene from other member(s) of the phylum, while originations are defined as the acquisition of a gene from members of other phyla outside the sampled genome set or by *de novo* gene formation.

Supplementary Data 5. arCOG copy number gain at duplication hotspots. The increase in copy number of particular arCOG families between the query genome reconstruction and its last ancestor. Letters in second column represent clusters of orthologous groups (COG) categories.

Supplementary Data 6. arCOG copy number loss at duplication hotspots. The decrease in copy number of particular arCOG families between the query genome reconstruction and its

last ancestor. Letters in second column represent clusters of orthologous groups (COG) categories.

Supplementary Data 7. arCOG annotations of originating gene families. Letters in second column represent clusters of orthologous groups (COG) categories.

Supplementary Data 8. Best hit taxonomy of originating gene families. Best results obtained by querying the protein family mediod against UniRef90 sequences with strain-level designations and excluding thaumarchaeotal matches.

Supplementary Data 9. Duplication and losses in originating and ancestral gene families. The percentage of gene families that have been duplicated or lost in gene families that have originated on LNS (Branch 305), LNS-2 (Branch 280) and prior to LNS (ancestral gene families).

Supplementary Data 10. Key pathways in Thaumarchaeota genomes and ancestral reconstructions. Describes the presence (black) or absence (white) of selected genes in ancestral reconstructions. Hashtag (#) indicates gene families that could not be confidently assigned to an ancestor by probabilistic reconstruction and so were determined using their presence/absence in extant genomes. Numbers in columns of extant genomes represent the copy number of the corresponding gene.

Supplementary Data 11. Functional gains in major evolutionary transitions. KEGG K numbers present in the stated descendant and absent in the stated ancestor.

Supplementary Data 12. Functional losses in major evolutionary transitions. KEGG K numbers absent in the stated descendant and present in the stated ancestor.

Supplementary Data 13. Best fitting model of core orthologs groups. The best fitting sequence evolution model for each partitioned gene used in the phylogenomic tree estimation in Figure 1.

Supplementary Data 14. Comparing different species tree estimations using gene tree-species tree reconciliation. The most likely species tree was predicted using the following tests: Log-likelihood difference (logL), approximate unbiased test (AU), bootstrap probability calculated from the multiscale bootstrap (NP), bootstrap probability calculated in the usual manner (BP), Kishino-Hasegawa test (KH), Shimodaira-Hasegawa test (SH), weighted Kishino-Hasegawa test (WKH) and weighted Shimodaira-Hasegawa test (SH).

Supplementary Data 15. Comparing different species tree estimations using constrained trees. The most likely species tree was predicted using the following tests: Log-likelihood difference (logL), logL difference from the maximal logl in the set (deltaL), bootstrap proportion using RELL method (RELL), Kishino-Hasegawa test (KH), Shimodaira-Hasegawa test (SH), weighted Kishino-Hasegawa test (WKH), weighted Shimodaira-Hasegawa test (WSH), expected likelihood weight (ELW), approximate unbiased test (AU). The last column indicates if a tree can be statistically rejected (Yes) or not (No).

Supplementary Data 16. Comparison of genome-predicted and experimentally determined optimal growth temperatures in Thaumarchaeota.
