# Supplementary material for: Gene duplication drives genome expansion in a major lineage of Thaumarchaeota
Source: Nat Commun. 2020 Oct 30;11:5494. doi: 10.1038/s41467-020-19132-x (PMC7603488; doi:10.1038/s41467-020-19132-x)
Supplement: Supplementary file 2 — Peer Review File [file 41467_2020_19132_MOESM2_ESM.pdf]

Reviewer #1 (Remarks to the Author):

The manuscript “Gene duplication drives genome expansion in Thaumarchaeota” by Sheridan et al. includes an in depth phylogenomic analysis of the phylum Thaumarchaeota with a specific focus on the order Nitrosophaerales. The authors demonstrate that (at least) two lateral gene transfer events to organisms of the Nitrosophaerales drastically shaped the gene repertoire of these organisms. Using publicly available genomes and newly reconstructed genomes from metagenomes, the authors demonstrate that gene duplication events were major drivers in the evolution of Nitrosophaerales.
I congratulate the authors for presenting a well-written and thoroughly thought through manuscript. I had read the manuscript before on bioRxiv and was very happy to be invited as a reviewer for this manuscript. However, before the paper can be recommended for publication, I have some minor and major comments that the authors should address:
- The title suggests gene duplications drives the genome expansion in the entire phylum but in fact the authors only looked at one of the many orders. I suggest rephrasing.
- The abstract is missing information in the function of genes that underwent duplication. This is in fact something that is addressed fairly late in the manuscript although it is so essential that it should be in the abstract.
- L33/34: change “microbial metabolism” to “microbial process”
- L57: “However, this study” should be “However, the aforementioned study” to avoid confusion
- L59: The authors should be more explicit what comparison the cited study included.
- Results are missing a paragraph on the retrieval of the new MAGs. I had to go to the supplementary to find out from which ecosystem the MAGs were retrieved. Although this seems minor, having a paragraph that explains the dataset and the new MAGs is essential for understanding the rest of the manuscript.
- L78-80: This sentence is contradicting itself. The authors say that it is broadly similar with an exception that both trees show poor support in the basal branches. I think I can guess from the supplementary information what the authors mean but it is as clear as mud the way it is written currently. Please rephrase.
- L82: The authors claim that previous classifications of Thaumarchaeota was solely based on amoA sequences. In fact, that is not true. There are few studies out there that use concatenated protein trees and also 16S rRNA genes. Please rephrase. (Remember, the proposal of the entire domain was based on protein alignemnts+trees!)
- I appreciate that the authors only compare their data to GTDB as I do not fully support GTDB as authority for naming bacteria and archaea. However, does the statement in L89-93 agree with GTDB analyses of the genomes?
- L97: Here I asked myself again – where do the MAGs come from?
- L109: What about contamination and strain heterogeneity?
- Important question: Could the detection of gene duplications come from strain heterogeneity of MAGs? I expect the authors to provide some evidence that this is not the case for their MAGs. Examples could include coverage analysis of scaffolds containing gene duplications, analysis of SAGs and cultured genomes, analysis of closed genomes. I think this could be one of the pitfalls of this study.
- L117: I think there was an issue with OGTs no working for certain temperature ranges. I think the authors should re-check this. I also don’t think that it adds much to the story as it is highly speculative due to the fact that e.g. Nitrososphaera gargensis (a thermophile) was not detected as such.
- L125-131: This is clear discussion part and no longer results. Generally, I do see frequent discussion parts in the results section. I’d appreciate if the authors could write a combined results and discussion section if the journal allows to do so.
- L137: “With the addition these new MAGs,…” – I think there is an “of” missing.
- L147-149: No results presented, sounds like an introduction.
- L195-201: It is unclear to the reader that this paragraph is the introduction to a detailed analysis starting in L202. The reader is left with a lot of questions in L196 and it is unclear that these are answered much later after a discussion of these findings takes place. Again, a combined results and

discussion section would help!
- L214-217: Discussion.
- L218: s_Bin19 – this is another reason why I think introducing the bins is so important. The reader has no idea where this term or this bin is coming from.
- L291: “more similar” than what? The comparison is missing.
- L261: “genes” should be “gene”
- Figure 1: Color legend is hard to understand as there are so many redundant colors. The image would drastically benefit from turning the tree about 30 degrees clockwise to have the labels of the rings readable in the middle.
- Figure 3: Is really hard to understand. I tried to understand it many times with reading the legend but I failed. It’s mainly due to the coloring probably. Please re-work this figure to convey the message it should.

Reviewer #2 (Remarks to the Author):

The manuscript “Gene duplication drives genome expansion in Thaumarchaeota” addresses the evolution of the phylum Thaumarchaeota based on genomes currently available, and focuses on the order Nitrososphaerales, based on 12 new metagenome-assembled genomes. Following extensive phylogenomic analyses, the study identified major mechanisms of genome evolution within the phylum, namely lateral gene transfers, and gene duplications and losses.

This is an interesting study that consolidates some previous observations and provides new insights into evolutionary patterns of a microbial phylum that plays a major role in biogeochemical cycling.

The manuscript is very well written, the analytical work is thorough and the results are generally well described. However, the study has some shortcomings when it comes to framing hypotheses and the relevance of the findings in light of previous knowledge, and do not clearly contextualizes results with previous observations, definitions and known taxonomy. There are also some inaccurate or over-statements that should be corrected or toned down. I urge the authors to be more conservative in their statements and acknowledge possible biases, especially in a study that is rooted in models. This will not take any value away from the study, but will rather make it more straightforward and lay better ground for further studies and hypotheses.

(1) Interesting as the results are, their implications and relevance in terms of general microbial evolution, or specific evolution and biology of Thaumarchaeota are unclear. With the exception of limited discussion, the manuscript provides little to no background or hypotheses to show the relevance and novelty of the core findings of the study, beyond the fact that it has not been done with these many thaumarchaeotal genomes before. For example: how interesting/unexpected/common are the major mechanisms of evolution identified, when compared to other archaeal or bacterial phyla? How do these patterns of gene duplication/loss fit in the bigger picture, in terms of prokaryotic/archaeal diversification and biology? Class Nitrososphaeria/AOA represent the most environmentally widespread archaea on the planet, despite their very specific metabolic niche – can this be interpreted in light of their evolutionary patterns (e.g. gene content and innovation)? What can be said about their niche differentiation and potential functional diversity, in light of previous hypotheses in the literature, especially between taxa with vast gene content/protein family differences?

(2) The references to thaumarchaeal lineages are very inconsistent throughout the manuscript, and hard to relate to previous designations. This is particularly true for the newly proposed families of Nitrososphaerales, which are central to the study, but remain largely anonymous, unless one digs through the supplementary table. The authors state in the methods that they adopted the nomenclature from a previous classification by Alves et al. 2018, although this was rarely done in the text. Given the congruency with amoA-based lineages, as stated by the authors, I strongly

recommend to establish clearly the relationships between family-level lineages described here and those defined by Alves et al., and use the nomenclature consistently throughout. Ideally, it would be very helpful to provide a supplementary figure matching the lineages between trees. Given that that amoA tree represents the most up-to-date phylogeny of all AOA lineages, this would allow to clearly identify the lineages discussed here and link them to the large amount of environmental and other metadata from previous research, summarized in that study. For example, there have been many observations and hypotheses regarding environmental niche and functional differences among Nitrososphaerales, and it would be extremely valuable to be able to link them to the results presented here. Moreover, several amoA-based lineages are largely underrepresented, or not represented at all in the case of putative Nitrosopumilales families, and this would allow to assess the data gaps. Again, contextualizing better the present results with previous information is not only essential to propagate new knowledge in the field, but it would also increase the value of the work done here. I have added further comments to the text below, also relating to taxonomic designations.

Comments on the text:

Line 14: AOA are not keystone "species"; rather "functional group", or "organisms". Also, keystone species has a more specific technical meaning, and shouldn't be used loosely. I suggest replacing it by a more general term, as suggested.

Line 15: There are no "six families" of Nitrososphaerales defined, but rather clades based on single marker genes. These six families seem to be proposed here based on the results of this study, so they should not be introduced as something that has been established. Refer instead to "major clades/lineages" until that is clarified in the results.

Lines 15-16: The way it reads now, it sounds like the major output of the study are the new genomes and then some evolutionary aspects were looked into. Similar to what I pointed out in comment (1), please state briefly the background leading to the central part of the study.

Line 33: AOA certainly have an important role in nitrogen cycling, but "one of the most important microbial metabolisms on the planet" is quite an overstatement. Please tone it town.

Lines 36-37: Specify that AOA represent class Nitrososphaeria, formally described in the Bergey's manual, and which includes all and only AOA, as far as characterized strains are concerned.

Lines 42-44: These lineages have been arbitrarily included in the Thaumarchaeota (which was defined based on AOA strains/genomics alone) based on suggestive phylogenetic branching, but as far as I am aware, there is no objective genomic or phenotypic evidence that they form a unified phylum with AOA, at the exclusion of Aigarchaeota or other closely related lineages. Since this is a widely propagated assumption, I believe the authors can nevertheless use it as point of reference. Nevertheless, please provide a reasonable argument to include them in the Thaumarchaeota in the current study and make the distinction clear here and throughout the manuscript as appropriate.

Lines 52-54: Same as pointed out in comment (1) above: why are these particular evolutionary mechanisms interesting, among the many evolutionary aspects that could have been addressed? What were they contrasted with? What is their biological significance and prevalence, based on what is known/hypothesized for Thaumarchaeota and other microbial taxa?

Lines 60-61: Same as above: why these mechanisms, and why are they particularly interesting in the case of Nitrososphaerales, so that it supports the relevance of the new genomes?

Line 63: Again, there are no six families of Nitrososphaerales defined up to this point. The proposal that these lineages known from single marker genes represent distinct families is a result of this study, so they should be referred to simply as lineages/clades up to the point where they are

proposed based on the results described, and contextualized with previous designations.

Line 76: Please write the full name of the branch support methods the first time they are mentioned; many readers may have no idea what "UF" and "SH-aLRT" mean. Also, Ultrafast Bootstrap is formally abbreviated to UFBoot.

Lines 77-78: What was compared were different marker gene selections/methods, not phylogenomic reconstruction methods, which is something else entirely. Please specify.

Lines 82-84: This is a misleading statement: phylogenetic analyses of amoA genes and subsequent classifications represented AOA, specifically their amoA gene evolution/taxonomy, and could have not been intended or interpreted as Thaumarchaeota classifications. While amoA classifications have been used as proxy for AOA organisms based on their congruency with 16S rRNA genes, they have always had separate classifications based on rRNA genes and genomes (1.1a/marine group I, 1.1b, etc). The same goes for non-AOA lineages, as seen by the fact that they actually have provisory names and have been tentatively included in Thaumarchaeota. This might be a good place to add some clarification regarding the comment on lines 42-44.

Lines 90-91: Please provide references for this statement (e.g. Bergey's Manual). The fact that Cenarchaeum spp. are phylogenetically placed within Nitrosopumilales, and closely related to Nitrosopumilus spp. within the order, has already been shown by, e.g. Alves et al. 2018 (doi.org/10.1038/s41467-018-03861-1), and others before. On the other hand, the observation that the Nitrosotaleales should be included in the order Nitrosopumilales, rather than representing its own order, is an important result, especially because it has been formally defined in the reference literature (Bergey's Manual). Formal taxonomic classifications are not traditionally defined based on ANI alone, but I strongly suggest that the authors include here a note about that, and perhaps a proposal to rectify the classification of Nitrosotaleales. If the comparative genomics criteria were used here used to define all these taxonomic ranks reported, including the new Nitrososphaerales families, then they should be consistently applied across the data.

Line 96: In "MY3", please name the organism and specify lineage, to make it relatable to previous literature.

Lines 100-105: These descriptions have been primarily defined based on environmental single marker genes, not isolates or genomes. Alves et al. 2018 (doi.org/10.1038/s41467-018-03861-1) has recently re-assessed the congruency of the marine groups, which referred to variable phylogenetic lineages across the literature. It would be best if the results here are linked to clear lineages with clear environmental associations, instead of reverting back to subjective designations.

Lines 103-108: The way this is written appears to imply that the number of genomes is of actual significance or somewhat unexpected. Rephrase it slightly to convey rather the disproportional overrepresentation of specific lineages, irrespective of their actual intra-diversity. Please mind that that has already been discussed by Alves et al. 2018.

Line 113: The greater genome sizes of Nitrososphaerales among AOA have already been reported in previous studies (e.g. Kerou et al. 2016, doi.org/10.1073/pnas.1601212113), which should be acknowledged. The additional genomes here confirm that observation, but should not be stated as a standalone or new finding.

Lines 114-116: The phylogenetic patterns of genomic GC content of AOA, including that of Cenarchaeum and other putative marine symbionts, have already been reported and discussed by Alves et al. 2018. Although this is a minor point in the whole story here, all results should be integrated with previous findings, not reported as novelty.

Line 128: “mesophiles in Nitrosocaldales” will surprise many readers. I suggest adding a bit more of information about the origin of theses genomes/organisms, and how closely related they actually are to thermophilic Nitrosocaldales.

Line 138: The Nitrososphaerales do not consist of “22 species, representing 9 genera”. Rather, the currently available genomes and new MAGs represent 22 species and 9 genera within Nitrososphaerales. There are likely many more species not represented, and possibly genera, as suggested by amoA gene diversity.

Lines 141-144: This is incorrectly stated: the splitting of these lineages has been strongly supported by previous amoA gene phylogenies - the new result here is the robust resolution of the basal relationships between those lineages.

Line 144: Is this entirely new, or is it already represented in previous single marker gene phylogenies, where it was placed within one of the other clades? Where is the respective amoA gene assigned to in previous classifications? It would be interesting to know whether it is actually a new lineage, or it could just represent a subclade underrepresented in the genomic dataset, and thus not reliably placed in relation to other lineages.

Lines 200-201: This statement is incorrect: genes for molybdate uptake and synthesis of molybdenum cofactor and molybdoenzymes have already been reported for Nitrosocosmicus spp. by Alves et al. 2019 (doi.org/10.3389/fmicb.2019.01571).

Line 203: Almost absent? What are the exceptions?

Lines 216-217: Provide reference for this statement.

Lines 232-234: This sentence is unclear. Please clarify.

Lines 250-254: This sentence is quite cryptic. Please rephrase it to something more interpretable in biological terms.

Line 261: Please do not make acronyms out of other acronyms (i.e. LA)… I understand the need for brevity, but use something like “AOA LCA”, in this case. The same applies to further abbreviations including “LCA”.

Lines 265-266: Since Nitrososphaeria is not used frequently throughout the manuscript, I suggest adding here “class Nitrososphaeria”.

Lines 342-343: This has also been reported for N. cavascurensis (Abby et al. 2018, doi.org/10.3389/fmicb.2018.00028).

Line 360: “Eutrophic” designates a rather extreme nutrient overload. I believe the authors mean “nutrient-rich”, or “higher nutrient availability”.

Line 367-368: Rephrase sentence to something less complicated, like “…but could help maintain respiratory…”.

Lines 391-394: It is unclear where this information comes from, and how it connects with the results. The information described about other archaeal lineages obviously comes from other study(ies), but the authors mention a “reassessment”. What reassessment was this and where was it performed? Was a citation forgotten somewhere in the sentence?

Lines 394-396: Related to my major comment (1) and the comment on the previously lines, the

manuscript does not provide sufficient background, or formulate hypotheses, that clearly substantiate the bold statement: "our analysis of thaumarchaeotal evolution may have uncovered a previously unappreciated paradigm of genome expansion with broader significance". Calling it a new paradigm is very ambitious without a more comprehensive context, and, the "broader significance" is quite obscure.

Line 404: Provide the date of final data collection.

Line 445-446: Bootstrap replicates, not "bootstraps". Also, the following statement is incorrect: the hill-climbing algorithm does not "further optimize branch support" – it is a component of IQ-TREE's tree search strategy to maximize tree likelihood.

Line 452: Specify what the -bb, -bnni, and -MFP parameters are.

Lines 478-479: This relates to my major comment (2) and other comments above – although the use of this nomenclature was stated here, it was actually rarely used throughout the text.

Lines 729-730: Was it rooted with both Aigarchaeota and Bathyarchaeota, as described in the methods, or just Bathyarchaeota?

Reviewer #3 (Remarks to the Author):

Major comments:
This manuscript by Sheridan et al. provides fundamental knowledge on the evolution of the Thaumarchaeota – suggesting the importance of gene duplication and other evolutionary processes in driving the genome expansion of Thaumarchaeota. Using 12 newly reconstructed MAGs, the authors expand on phylogenetic and metabolic traits associated with a specific order, Nitrososphaerales. The different fates of gene families indicate the potential niche-specificity of these strains in adapting the specific environments. This discovery of evolutionary events in the evolution history of Nitrososphaerales is a fundamental finding by the genome-based inference. Overall, I think this manuscript is well-written and has sophisticated, well-self-supported, and careful evolutionary analyses. I think the gene duplication revealed by this manuscript is quite convincing. However, I do have some concerns about the scope and applicability of the overall findings and premise, and some of the methods. In particular, I am not convinced that these observations currently are robust enough at the resolution of the Nitrososphaerales order as a whole to be extrapolated to Thaumarchaea, let alone all Archaea.

Given the presence of few genomes for a number of orders, I am not sure the primary findings on Nitrososphaerales can necessarily be extrapolated to Thaumarchaeaota as a whole. I would suggest the authors to think further and bolster this argument potentially by using higher-quality genomes (45% completeness is too low and practically unacceptable), and by reconstructing additional genomes from these new orders from publicly available metagenomes. Low completeness is okay if the study only investigates "identified proteins" and presents no interest in "missing proteins" but that is not the case with evolutionary analyses.

I did not get a clear idea of gene duplication links to functional gain. Gene duplication/loss are followed by gene origination for Nitrososphaerales. All the gene content analyses reflect the metabolic trait difference of Nitrososphaerales from other marine groups. This is also the way the authors analyze other groups, like LNC, LNS, and LNP. It seems that LGT or gene formation may be more responsible for niche-specific adaptions. I suggest the authors pay more emphasis on illustrating functional contributions and mechanisms between gene duplication and LGT.

The results describing diversity of specific thaumarchaea groups (like for example the deep water

group) are predicated on sequenced genomes that may not represent the true diversity of these groups. Can this be normalized to account for any biases?

I see no discussion of the number of gene duplications observed here and their comparison to observed numbers in other archaeal/bacterial phyla. A comparison would help illustrate the findings in better context.

Lines 235-237 – Do the estimates of gene duplication take into account the uneven number of genomes across the different orders?

As the author pointed out in the discussion, "the predominant mode of genome expansion was gene duplication of both ancestral and originating gene families, with higher duplication rates occurring in the latter", I agree that this is well-supported by the evolutionary analysis but I did not see a clear explanation and detailed mechanistic interpretation. Any new ideas or comparisons to previous observations would be truly meaningful.

Methods: I think a 45% genome completeness cutoff used for evaluating changes in protein content could introduce significant bias? Could genes missing due to incomplete genomes be interpreted as lost?

Minor comments:
I suggest to label the three of the six known families that have the first genomes in the Fig. 1 tree. Meanwhile, it will also be better to label the Nitrososphaerales clade in the tree.

Please clarify the importance of the newly discovered 12 MAGs in assisting the whole phylum level evolutionary analysis. The contribution of the well-established Nitrososphaerales genome collection to the evolutionary analysis should be also highlighted. Else, this seems like revisiting Thaumarchaeota evolutionary histories.

Gene duplication is mentioned in the context of increasing gene numbers and genome size in each genome, that makes sense. I however, read proteome expansion to imply functional expansion. But is this true? I think clarifying this one way or another would be useful

Line 274, when new genes are gained, you do not know whether it is from inter-LGT or de novo gene formation. So gene origination doesn't equal to LGT.

Line 277, do you mean that if these gene families have homologs in larger databases, they are more likely to be from LGT events? But how about it is due to its LGT to other groups?

Overall, I suggest the authors to label each figures to make them easier to follow. They are not particularly reader friendly.

Technical problems:
Line 132. The period is not necessary.
Line 152-154. Add citation to the early-acquired traits.

**RESPONSE TO REVIEWER COMMENTS**

We thank the three reviewers for their constructive comments, which improve the manuscript. The line numbering refers to the revised manuscript without track change.

Reviewer #1 (Remarks to the Author):

The manuscript "Gene duplication drives genome expansion in Thaumarchaeota" by Sheridan et al. includes an in depth phylogenomic analysis of the phylum Thaumarchaeota with a specific focus on the order Nitrosophaerales. The authors demonstrate that (at least) two lateral gene transfer events to organisms of the Nitrosophaerales drastically shaped the gene repertoire of these organisms. Using publicly available genomes and newly reconstructed genomes from metagenomes, the authors demonstrate that gene duplication events were major drivers in the evolution of Nitrosophaerales.
I congratulate the authors for presenting a well-written and thoroughly thought through manuscript. I had read the manuscript before on bioRxiv and was very happy to be invited as a reviewer for this manuscript. However, before the paper can be recommended for publication, I have some minor and major comments that the authors should address:

- The title suggests gene duplications drives the genome expansion in the entire phylum but in fact the authors only looked at one of the many orders. I suggest rephrasing.
We thank the reviewer for their suggestion and have modified the title.

- The abstract is missing information in the function of genes that underwent duplication. This is in fact something that is addressed fairly late in the manuscript although it is so essential that it should be in the abstract.
Some information has now been added (ln 26-28).

- L33/34: change "microbial metabolism" to "microbial process"
This has now been changed (ln 37).

- L57: "However, this study" should be "However, the aforementioned study" to avoid confusion
This has now been changed (ln 68-69).

- L59: The authors should be more explicit what comparison the cited study included.
More explicit detail has now been added (ln 69-73).

- Results are missing a paragraph on the retrieval of the new MAGs. I had to go to the supplementary to find out from which ecosystem the MAGs were retrieved. Although this seems minor, having a paragraph that explains the dataset and the new MAGs is essential for understanding the rest of the manuscript.
A paragraph has now been added in the method section (ln 447-462) of the main manuscript and a sentence has also been added in the result section (ln 85-87).

- L78-80: This sentence is contradicting itself. The authors say that it is broadly similar with an exception that both trees show poor support in the basal branches. I think I can guess from the supplementary information what the authors mean but it is as clear as mud the way it is written currently. Please rephrase.
This has now been rephrased (ln 93-95).

- L82: The authors claim that previous classifications of Thaumarchaeota was solely based on amoA sequences. In fact, that is not true. There are few studies out there that use concatenated protein trees and also 16S rRNA genes. Please rephrase. (Remember, the proposal of the entire domain was based on protein alignemnts+trees!)
This has now been rephrased (ln 95-100).

- I appreciate that the authors only compare their data to GTDB as I do not fully support GTDB as authority for naming bacteria and archaea. However, does the statement in L89-93 agree with GTDB analyses of the genomes?
We agree with the reviewer that GTDB is not the authority for naming bacteria and archaea and our classification differs from the GTDB-tk classification , as shown by the comparison between table S2 and S3. We have now ordered the genomes in the GTDB-tk classification (Table S3) in the same order as in our classification (Table S2) to make it easier for the reader to compare differences.

- L97: Here I asked myself again – where do the MAGs come from?
A sentence has been added (ln 85-87). In addition, the environmental source has been added (ln 113).

- L109: What about contamination and strain heterogeneity?
A sentence on contamination and strain heterogeneity has now been added to the text and linked to Table S1 where all detailed values are indicated (ln 136-137).

- Important question: Could the detection of gene duplications come from strain heterogeneity of MAGs? I expect the authors to provide some evidence that this is not the case for their MAGs. Examples could include coverage analysis of scaffolds containing gene duplications, analysis of SAGs and cultured genomes, analysis of closed genomes. I think this could be one of the pitfalls of this study.
We thank the reviewer for raising this important point and have now developed a section in the supplementary information (Supplementary Information: Effect of MAG contamination on gene duplication estimates) to provide evidence that gene duplications likely do not come from strain heterogeneity of MAGs. In short, we present two main lines of evidence:

1) While strain heterogeneity might inflate estimates of duplications on the terminal branches of the tree (by creating spurious lineage-specific duplicates in gene trees), the largest duplication events (indicated by nodes A, B, C and D in Figure 3) occur in non-terminal branches of the tree. This provides evidence that these deep duplications come from gene tree patterns in which the duplication is ancestral to an entire group of taxa, and thus these duplications are unlikely to be generated by strain heterogeneity. Note also that the second largest duplication event (node D in Figure 3) occurs ancestrally to a clade containing one MAG and three nearly complete genomes from cultured organisms, each with 0% strain heterogeneity. The percentage of gene families than underwent duplication is similar between the 4 individual genomes of this clade (Table S9), suggesting these inferences are not the result of strain heterogeneity.
2) Non-metric multi-dimensional scaling of the coverage profiles of duplicated and non-duplicated genes in TH1173 and TH5895 (MAG descendants of the two largest duplication hotspots) revealed that the majority of duplicated genes (67% for TH1173 and 80% for TH5895) are within the 50% quantile of all genes (Figure S9), further indicating that duplicate genes are unlikely to be the result of strain heterogeneity.

- L117: I think there was an issue with OGTs no working for certain temperature ranges. I think the authors should re-check this. I also don't think that it adds much to the story as it is highly

speculative due to the fact that e.g. Nitrososphaera gargensis (a thermophile) was not detected as such.

We agree that in silico OGT predictions seem to be more accurate for mesophilic than thermophilic organisms, if the experimental measurements are correct. As shown in Table S16 and discussed in the supplementary text “Extended optimal growth temperature”, we have tempered our interpretations based on these limitations.

In regard to *Nitrososphaera gargensis*, it is worth noting that the experimentally predicted OGT is 46 °C, only 4 °C more than the related mesophile Nitrososphaera viennensis (Table S16), so it is perhaps not very surprising that they have similar in silico predictions.

While development of such approaches is progressing fast, they haven’t yet been applied to a large range of thaumarchaeotal genomes. Published studies (such as de la Torre et al., 2008; Hua et al., 2018) suggested that the ancestor of all AOA was a thermophile, while our study suggests that the common ancestor of AOA may have been a mesophile and that the hot origin of Thaumarchaeota happened earlier in their evolutionary history. We believe that this important difference merits inclusion of the OGT section in this manuscript, especially as it was well appreciated by Reviewer 2.

- L125-131: This is clear discussion part and no longer results. Generally, I do see frequent discussion parts in the results section. I’d appreciate if the authors could write a combined results and discussion section if the journal allows to do so.
The journal does not allow an integrated results-discussion section. However, we think that some additional context in the results section is appropriate and beneficial to the reader as this article covers several specialist concepts of evolution and metabolism.

- L137: “With the addition these new MAGs,…” – I think there is an “of” missing.
This sentence has been modified (ln 122).

- L147-149: No results presented, sounds like an introduction.
We agree with this comment, but this sentence is an introduction to the paragraph and we would prefer to keep this if possible.

- L195-201: It is unclear to the reader that this paragraph is the introduction to a detailed analysis starting in L202. The reader is left with a lot of questions in L196 and it is unclear that these are answered much later after a discussion of these findings takes place. Again, a combined results and discussion section would help!
We have deleted some introductory sentences from this paragraph to improve its readability.

- L214-217: Discussion.
We appreciate the earlier suggestion of a merged result and discussion section, but the journal does not allow it.

- L218: s_Bin19 – this is another reason why I think introducing the bins is so important. The reader has no idea where this term or this bin is coming from.
Information on the environmental source and the fact that it is a MAG has been added (ln 242).

- L291: “more similar” than what? The comparison is missing.
This has now been added (ln 243-245).

- L261: “genes” should be “gene”
This has been corrected (ln 284).

- Figure 1: Color legend is hard to understand as there are so many redundant colors. The image would drastically benefit from turning the tree about 30 degrees clockwise to have the labels of the rings readable in the middle.
The figure has been turned clockwise to put ring labels in the middle. In regard to the colours, we have darkened the colours of some of the order-level clades to reduce colour similarity to other clades. Additionally, the "Completeness" ring band is now placed between the "Genome type" and "Environment" ring bands to improve discrimination between these bands. Finally, the legends now include colour gradient rather than upper and lower limits to improve the readability of the values. We have also added a reference to Table S1 on the legend, which details the numerical information used to produce the ring bands.

- Figure 3: Is really hard to understand. I tried to understand it many times with reading the legend but I failed. It's mainly due to the coloring probably. Please re-work this figure to convey the message it should.
We are sorry that this figure was unclear to the reviewer. To address this, we have heavily modified the legend, added lines to highlight the area of the tree containing the main hotspots of duplication and loss and clarified the main branches discussed in the text. Additionally, we have labelled the scales and modified and labelled the bar indicating the taxonomy of the tree leaves. Finally, we have also reworded the legend to facilitate its understanding.

## Reviewer #2 (Remarks to the Author):

The manuscript "Gene duplication drives genome expansion in Thaumarchaeota" addresses the evolution of the phylum Thaumarchaeota based on genomes currently available, and focuses on the order Nitrososphaerales, based on 12 new metagenome-assembled genomes. Following extensive phylogenomic analyses, the study identified major mechanisms of genome evolution within the phylum, namely lateral gene transfers, and gene duplications and losses.

This is an interesting study that consolidates some previous observations and provides new insights into evolutionary patterns of a microbial phylum that plays a major role in biogeochemical cycling.

The manuscript is very well written, the analytical work is thorough and the results are generally well described. However, the study has some shortcomings when it comes to framing hypotheses and the relevance of the findings in light of previous knowledge, and do not clearly contextualizes results with previous observations, definitions and known taxonomy. There are also some inaccurate or over-statements that should be corrected or toned down. I urge the authors to be more conservative in their statements and acknowledge possible biases, especially in a study that is rooted in models. This will not take any value away from the study, but will rather make it more straightforward and lay better ground for further studies and hypotheses.
We thank the reviewer for this positive assessment of the manuscript and for those constructive comments. We have carefully revised the manuscript by bringing lots of attention to integrating all our findings in light of previous knowledge and have corrected all inaccuracies or overstatements (see detail below).

(1) Interesting as the results are, their implications and relevance in terms of general microbial evolution, or specific evolution and biology of Thaumarchaeota are unclear. With the exception of limited discussion, the manuscript provides little to no background or hypotheses to show the relevance and novelty of the core findings of the study, beyond the fact that it has not been done with these many thaumarchaeotal genomes before. For example:

how interesting/unexpected/common are the major mechanisms of evolution identified, when compared to other archaeal or bacterial phyla?
We have now clarified the hypothesis and associated relevant background of this study in the introduction (ln 54-65). In addition, we have complemented the discussion to compare those findings to previous knowledge on other archaeal and bacterial phyla (ln 429-436).

How do these patterns of gene duplication/loss fit in the bigger picture, in terms of prokaryotic/archaeal diversification and biology?
Research on gene duplication in prokaryotes, especially archaea, is relatively limited and a thorough independent study including a larger number of microbial phyla would be required. We are planning to perform such study, but such approach would be too intensive to be included in the present manuscript.

Class Nitrososphaeria/AOA represent the most environmentally widespread archaea on the planet, despite their very specific metabolic niche – can this be interpreted in light of their evolutionary patterns (e.g. gene content and innovation)? What can be said about their niche differentiation and potential functional diversity, in light of previous hypotheses in the literature, especially between taxa with vast gene content/protein family differences?
While this aspect is approached in the results, we acknowledge that this was missing from the discussion and have added a few sentences to highlight this important point (ln 404-410).

(2) The references to thaumarchaeal lineages are very inconsistent throughout the manuscript, and hard to relate to previous designations. This is particularly true for the newly proposed families of Nitrososphaerales, which are central to the study, but remain largely anonymous, unless one digs through the supplementary table. The authors state in the methods that they adopted the nomenclature from a previous classification by Alves et al. 2018, although this was rarely done in the text. Given the congruency with amoA-based lineages, as stated by the authors, I strongly recommend to establish clearly the relationships between family-level lineages described here and those defined by Alves et al., and use the nomenclature consistently throughout.
Ideally, it would be very helpful to provide a supplementary figure matching the lineages between trees. Given that that amoA tree represents the most up-to-date phylogeny of all AOA lineages, this would allow to clearly identify the lineages discussed here and link them to the large amount of environmental and other metadata from previous research, summarized in that study. For example, there have been many observations and hypotheses regarding environmental niche and functional differences among Nitrososphaerales, and it would be extremely valuable to be able to link them to the results presented here. Moreover, several amoA-based lineages are largely underrepresented, or not represented at all in the case of putative Nitrosopumilales families, and this would allow to assess the data gaps. Again, contextualizing better the present results with previous information is not only essential to propagate new knowledge in the field, but it would also increase the value of the work done here. I have added further comments to the text below, also relating to taxonomic designations.
We regret that that the reviewer had to dig into our supplementary material in order to link the novel genomes to the most up-to-date phylogeny of AOA and associated environmental data. We have therefore amended the nomenclature in the text by adding the family-level lineages described by Alves. In addition, we have added a supplementary figure (Figure S1)  placing the genomes used in this study on the *amoA* phylogenetic tree described by Alves et al.

Comments on the text:

Line 14: AOA are not keystone “species”; rather “functional group”, or “organisms”. Also, keystone

species has a more specific technical meaning, and shouldn't be used loosely. I suggest replacing it by a more general term, as suggested.
This has now been changed (ln 18).

Line 15: There are no "six families" of Nitrososphaerales defined, but rather clades based on single marker genes. These six families seem to be proposed here based on the results of this study, so they should not be introduced as something that has been established. Refer instead to "major clades/lineages" until that is clarified in the results.
This has now been changed (ln 20).

Lines 15-16: The way it reads now, it sounds like the major output of the study are the new genomes and then some evolutionary aspects were looked into. Similar to what I pointed out in comment (1), please state briefly the background leading to the central part of the study.
The abstract has been modified to highlight the main objective of the study (ln 20-22).

Line 33: AOA certainly have an important role in nitrogen cycling, but "one of the most important microbial metabolisms on the planet" is quite an overstatement. Please tone it town.
This has been toned down (ln 37-40).

Lines 36-37: Specify that AOA represent class Nitrososphaeria, formally described in the Bergey's manual, and which includes all and only AOA, as far as characterized strains are concerned.
This has been amended and the Bergey's reference has been added (ln 41-43).

Lines 42-44: These lineages have been arbitrarily included in the Thaumarchaeota (which was defined based on AOA strains/genomics alone) based on suggestive phylogenetic branching, but as far as I am aware, there is no objective genomic or phenotypic evidence that they form a unified phylum with AOA, at the exclusion of Aigarchaeota or other closely related lineages. Since this is a widely propagated assumption, I believe the authors can nevertheless use it as point of reference. Nevertheless, please provide a reasonable argument to include them in the Thaumarchaeota in the current study and make the distinction clear here and throughout the manuscript as appropriate.
The classification of these non-AOA archaea as Thaumarchaeota indeed arises from their monophyletic affiliation. As mentioned by the reviewer, almost all studies referring to these organisms, including the first culture (Kato et al., 2019), all available metagenomes (references in the paper) as well as environmental studies on these organisms (for example Weber et al., 2015; 2019) refer to them as Thaumarchaeota. There have also been attempts in recent years to establish unified thresholds for phyla in the archaeal tree of life, but at present there is no conclusive answer and this is well beyond the remit of this work. We have however now specified that we consider both AOA and non-AOA lineages in the phylum Thaumarchaeota in Material and Methods (ln 464) and we also added another sentence (as suggested by another comment below) in the result section (ln 99-100) to highlight the lack of unifying metabolism at the phylum level.

Lines 52-54: Same as pointed out in comment (1) above: why are these particular evolutionary mechanisms interesting, among the many evolutionary aspects that could have been addressed? What were they contrasted with? What is their biological significance and prevalence, based on what is known/hypothesized for Thaumarchaeota and other microbial taxa?
As developed in response to comment 1, an entire section has been added in the introduction (ln 54-65) to clarify the hypothetical mechanisms suggested ahead of the study, based on the previous knowledge. As described in the revised discussion, duplication and LGT have been described as the key evolutionary mechanisms in eukaryote and prokaryote evolution, respectively, so these findings have high biological relevance (ln 442-444).

Lines 60-61: Same as above: why these mechanisms, and why are they particularly interesting in the case of Nitrososphaerales, so that it supports the relevance of the new genomes?
This is now explained in the introduction in (ln 54-65) and in the discussion (ln 443-445).

Line 63: Again, there are no six families of Nitrososphaerales defined up to this point. The proposal that these lineages known from single marker genes represent distinct families is a result of this study, so they should be referred to simply as lineages/clades up to the point where they are proposed based on the results described, and contextualized with previous designations.
This has been modified throughout.

Line 76: Please write the full name of the branch support methods the first time they are mentioned; many readers may have no idea what "UF" and "SH-aLRT" mean. Also, Ultrafast Bootstrap is formally abbreviated to UFBoot.
This has been amended throughout.

Lines 77-78: What was compared were different marker gene selections/methods, not phylogenomic reconstruction methods, which is something else entirely. Please specify.
This has been amended (ln 92-93).

Lines 82-84: This is a misleading statement: phylogenetic analyses of amoA genes and subsequent classifications represented AOA, specifically their amoA gene evolution/taxonomy, and could have not been intended or interpreted as Thaumarchaeota classifications. While amoA classifications have been used as proxy for AOA organisms based on their congruency with 16S rRNA genes, they have always had separate classifications based on rRNA genes and genomes (1.1a/marine group I, 1.1b, etc). The same goes for non-AOA lineages, as seen by the fact that they actually have provisory names and have been tentatively included in Thaumarchaeota. This might be a good place to add some clarification regarding the comment on lines 42-44.
This has been amended (ln 95-100).

Lines 90-91: Please provide references for this statement (e.g. Bergey's Manual). The fact that Cenarchaeum spp. are phylogenetically placed within Nitrosopumilales, and closely related to Nitrosopumilus spp. within the order, has already been shown by, e.g. Alves et al. 2018 (doi.org/10.1038/s41467-018-03861-1), and others before. On the other hand, the observation that the Nitrosotaleales should be included in the order Nitrosopumilales, rather than representing its own order, is an important result, especially because it has been formally defined in the reference literature (Bergey's Manual). Formal taxonomic classifications are not traditionally defined based on ANI alone, but I strongly suggest that the authors include here a note about that, and perhaps a proposal to rectify the classification of Nitrosotaleales. If the comparative genomics criteria were used here used to define all these taxonomic ranks reported, including the new Nitrososphaerales families, then they should be consistently applied across the data.
The comment about Cenarchaeum spp reclassification has been removed and a reference has been added for the assignment of Ca. Nitrosotalea into their own order. Additionally we have added a note suggesting that the validity of the Ca. Nitrosotaleales order may need to be reconsidered (ln 106-108). In regard to the genomics-based taxonomic ranks, the criteria are indeed consistently applied across the data.

Line 96: In "MY3", please name the organism and specify lineage, to make it relatable to previous literature.
This has been added (ln 114-115).

Lines 100-105: These descriptions have been primarily defined based on environmental single

marker genes, not isolates or genomes. Alves et al. 2018 (doi.org/10.1038/s41467-018-03861-1) has recently re-assessed the congruency of the marine groups, which referred to variable phylogenetic lineages across the literature. It would be best if the results here are linked to clear lineages with clear environmental associations, instead of reverting back to subjective designations.
We have inserted the lineages from Alves but also kept the broad environmental associations for readers less familiar with thaumarchaeotal phylogenetic classification (as published in Ren et al., ISME, 2019) (ln 123-131).

Lines 103-108: The way this is written appears to imply that the number of genomes is of actual significance or somewhat unexpected. Rephrase it slightly to convey rather the disproportional overrepresentation of specific lineages, irrespective of their actual intra-diversity. Please mind that that has already been discussed by Alves et al. 2018.
This has now been added in the manuscript (ln 118-123).

Line 113: The greater genome sizes of Nitrososphaerales among AOA have already been reported in previous studies (e.g. Kerou et al. 2016, doi.org/10.1073/pnas.1601212113), which should be acknowledged. The additional genomes here confirm that observation, but should not be stated as a standalone or new finding.
This has been amended (ln 138-139).

Lines 114-116: The phylogenetic patterns of genomic GC content of AOA, including that of Cenarchaeum and other putative marine symbionts, have already been reported and discussed by Alves et al. 2018. Although this is a minor point in the whole story here, all results should be integrated with previous findings, not reported as novelty.
This has been amended (ln 141).

Line 128: “mesophiles in Nitrosocaldales” will surprise many readers. I suggest adding a bit more of information about the origin of theses genomes/organisms, and how closely related they actually are to thermophilic Nitrosocaldales.
This has been amended and the *amoA* amino acid identity of these organisms to the thermophile *Ca*. Nitrosocaldus islandicus 3F has been added (ln 154-155).

Line 138: The Nitrososphaerales do not consist of “22 species, representing 9 genera”. Rather, the currently available genomes and new MAGs represent 22 species and 9 genera within Nitrososphaerales. There are likely many more species not represented, and possibly genera, as suggested by amoA gene diversity.
This has been amended (ln 165-166).

Lines 141-144: This is incorrectly stated: the splitting of these lineages has been strongly supported by previous amoA gene phylogenies - the new result here is the robust resolution of the basal relationships between those lineages.
The sentence has been modified to correct this point (ln 169-171).

Line 144: Is this entirely new, or is it already represented in previous single marker gene phylogenies, where it was placed within one of the other clades? Where is the respective amoA gene assigned to in previous classifications? It would be interesting to know whether it is actually a new lineage, or it could just represent a subclade underrepresented in the genomic dataset, and thus not reliably placed in relation to other lineages.
Unfortunately, neither an *amoA* or 16S rRNA gene could be detected in this MAG, so it is difficult to predict its exact phylogenetic placement. This is now stated in the text (ln 173-174). More genomes will be required to assess this conclusively.

Lines 200-201: This statement is incorrect: genes for molybdate uptake and synthesis of molybdenum cofactor and molybdoenzymes have already been reported for Nitrosocosmicus spp. by Alves et al. 2019 (doi.org/10.3389/fmicb.2019.01571).
This has been amended (ln 223-224).

Line 203: Almost absent? What are the exceptions?
This information has been added (ln 226-228).

Lines 216-217: Provide reference for this statement.
Reference added (ln 240).

Lines 232-234: This sentence is unclear. Please clarify.
The sentence has been modified (ln 256-257).

Lines 250-254: This sentence is quite cryptic. Please rephrase it to something more interpretable in biological terms.
The sentence has been modified and now present the predicted biological function of the arCOGs (ln 274-277).

Line 261: Please do not make acronyms out of other acronyms (i.e. LA)… I understand the need for brevity, but use something like "AOA LCA", in this case. The same applies to further abbreviations including "LCA".
This has been amended throughout.

Lines 265-266: Since Nitrososphaeria is not used frequently throughout the manuscript, I suggest adding here "class Nitrososphaeria".
This has been added (ln 290-291).

Lines 342-343: This has also been reported for N. cavascurensis (Abby et al. 2018, doi.org/10.3389/fmicb.2018.00028).
This has been added (ln 368).

Line 360: "Eutrophic" designates a rather extreme nutrient overload. I believe the authors mean "nutrient-rich", or "higher nutrient availability".
This has been amended (ln 384).

Line 367-368: Rephrase sentence to something less complicated, like "…but could help maintain respiratory…".
This has been amended (ln 391-392).

Lines 391-394: It is unclear where this information comes from, and how it connects with the results. The information described about other archaeal lineages obviously comes from other study(ies), but the authors mention a "reassessment". What reassessment was this and where was it performed? Was a citation forgotten somewhere in the sentence?
We agree that "reassessment" was a poor choice of word. "reinterpretation" is less ambiguous. We have also made it clearer that we are using the results of a previous study and have connected it more explicitly with our results (ln 431-438).

Lines 394-396: Related to my major comment (1) and the comment on the previously lines, the manuscript does not provide sufficient background, or formulate hypotheses, that clearly

substantiate the bold statement: “our analysis of thaumarchaeotal evolution may have uncovered a previously unappreciated paradigm of genome expansion with broader significance”. Calling it a new paradigm is very ambitious without a more comprehensive context, and, the “broader significance” is quite obscure.
We have added a few sentences throughout the manuscript to clarify the paradigm-shifting view and the broader significance of our results.

Line 404: Provide the date of final data collection.
This has been added (ln 466).

Line 445-446: Bootstrap replicates, not “bootstraps”. Also, the following statement is incorrect: the hill-climbing algorithm does not “further optimize branch support” – it is a component of IQ-TREE’s tree search strategy to maximize tree likelihood.
These have been rephrased (ln 504-506).

Line 452: Specify what the -bb, -bnni, and -MFP parameters are.
This has been amended (ln 512-513).

Lines 478-479: This relates to my major comment (2) and other comments above – although the use of this nomenclature was stated here, it was actually rarely used throughout the text.
We have now added the Alves nomenclature in several places of the manuscript and also added the classifications to the coloured branches in Figure 2. In addition, all AOA genomes with an *amoA* gene were compared to the Alves classification in Table S2 and in Figure S1.

Lines 729-730: Was it rooted with both Aigarchaeota and Bathyarchaeota, as described in the methods, or just Bathyarchaeota?
Phylogenomic trees were rooted with the Bathyarchaeota genomes. This has been amended in the methods (ln 506).

Reviewer #3 (Remarks to the Author):

Major comments:
This manuscript by Sheridan et al. provides fundamental knowledge on the evolution of the Thaumarchaeota – suggesting the importance of gene duplication and other evolutionary processes in driving the genome expansion of Thaumarchaeota. Using 12 newly reconstructed MAGs, the authors expand on phylogenetic and metabolic traits associated with a specific order, Nitrososphaerales. The different fates of gene families indicate the potential niche-specificity of these strains in adapting the specific environments. This discovery of evolutionary events in the evolution history of Nitrososphaerales is a fundamental finding by the genome-based inference. Overall, I think this manuscript is well-written and has sophisticated, well-self-supported, and careful evolutionary analyses. I think the gene duplication revealed by this manuscript is quite convincing. However, I do have some concerns about the scope and applicability of the overall findings and premise, and some of the methods. In particular, I am not convinced that these observations currently are robust enough at the resolution of the Nitrososphaerales order as a whole to be extrapolated to Thaumarchaea, let alone all Archaea.

Given the presence of few genomes for a number of orders, I am not sure the primary findings on Nitrososphaerales can necessarily be extrapolated to Thaumarchaeaota as a whole.
We have revised the title and the discussion to emphasise that our results were established for Nitrososphaerales. In addition, we have clarified findings for other archaeal lineages (as stated in ln

429-436) based on a reinterpretation of previous results. We also highlighted to need of further analyses to other microbial lineages.

I would suggest the authors to think further and bolster this argument potentially by using higher-quality genomes (45% completeness is too low and practically unacceptable), and by reconstructing additional genomes from these new orders from publicly available metagenomes. Low completeness is okay if the study only investigates “identified proteins” and presents no interest in “missing proteins” but that is not the case with evolutionary analyses.

Concerning the low completeness of genomes used, the gene-tree species-tree reconciliation method we used, Amalgamated Likelihood Estimation, incorporates estimates of genome completeness into the analysis (estimation of gene loss rates) directly, so inclusion of 45% completeness genomes seems suitable for this approach. In regard to our main findings, it is also worth noting that the major hotspots of duplication and loss occurred for a clade where genomes have high completeness (average of 91.1%).

Concerning the reconstruction of additional genomes from these new orders, we first would like to stress out the novelty (and high completeness) of these genome reconstructions despite the existence of an active community working on Thaumarchaeota. While more high-quality genomes are always better, we do not think that mining for additional genomes from public databases would alter the major findings of this work. Considering the computational expense of metagenome assembly and also considering that many MAGs have already been binned by the metagenome submitter lab or by other labs mining public metagenomes for MAGs (such as the creators of the UBA MAGs), this would likely not be an efficient strategy for obtaining new Nitrososphaerales genomes. Nonetheless, we are part a large consortium (CLUE-TERRA) aiming to reconstruct and analyse additional genomes from 6,000 publicly available metagenomes, but this analysis is currently ongoing, and the full results will not be available until the end of 2020 at the earliest. Analysis of 500 of these metagenomes is already complete but did not contain any additional lineages affiliated to Thaumarchaeota. This is likely due to the fact that Thaumarchaeota are generally present at very low abundance in many terrestrial environments (< 1 %), resulting in a very low recovery rate of Thaumarchaeota MAGs. Therefore, as performed in our study, either deep-sequencing or co-assembly of a large number of metagenomes is required to recover a small number of AOA genomes.

I did not get a clear idea of gene duplication links to functional gain. Gene duplication/loss are followed by gene origination for Nitrososphaerales. All the gene content analyses reflect the metabolic trait difference of Nitrososphaerales from other marine groups. This is also the way the authors analyze other groups, like LNC, LNS, and LNP. It seems that LGT or gene formation may be more responsible for niche-specific adaptions. I suggest the authors pay more emphasis on illustrating functional contributions and mechanisms between gene duplication and LGT.
A section has now been added to discuss the relative contribution of origination and duplication for functional gain in the discussion (ln 422-427).

The results describing diversity of specific thaumarchaea groups (like for example the deep water group) are predicated on sequenced genomes that may not represent the true diversity of these groups. Can this be normalized to account for any biases?
We believe the reviewer refers to the section in L102-108 of the original manuscript. We agree that the genomes do not represent the full diversity of the Thaumarchaeota. This section was intended to convey that point and highlight gaps in genome representation. We have now modified this section (ln 118-133) to clarify this and created an additional supplementary figure (Figure S1) to further clarify this point.

I see no discussion of the number of gene duplications observed here and their comparison to

observed numbers in other archaeal/bacterial phyla. A comparison would help illustrate the findings in better context.
The comparison with other archaeal lineages is mentioned in the discussion based on a previous study (Williams et al., 2017, PNAS). We have modified the discussion to give an indication of the scale of the origination and duplication events (ln 434). This seems more appropriate than adding exact numbers given that the Williams study was based on a small number of representatives from a range of phyla, rather than a targeted examination of a single phylum as we present here.

Lines 235-237 – Do the estimates of gene duplication take into account the uneven number of genomes across the different orders?
The estimates of gene duplication are based on reconciliation between species and gene trees, so the uneven number of genomes across the different orders is indeed taken into account.

As the author pointed out in the discussion, “the predominant mode of genome expansion was gene duplication of both ancestral and originating gene families, with higher duplication rates occurring in the latter”, I agree that this is well-supported by the evolutionary analysis but I did not see a clear explanation and detailed mechanistic interpretation. Any new ideas or comparisons to previous observations would be truly meaningful.
We believe that our approach allows us to support this observation but unfortunately does not allow us to provide evidence for a mechanism. In our detailed analysis of the duplications in TH1173 and TH5895 we did observed that many duplicated genes are next to each other on the genome, indicating that unequal crossing over is the mechanism of these duplications. However, developing tools to quantify this in extant and ancestral genome reconstructions is beyond the scope of this work.

Methods: I think a 45% genome completeness cutoff used for evaluating changes in protein content could introduce significant bias? Could genes missing due to incomplete genomes be interpreted as lost?
This 45% completeness has been accounted for probabilistically using ALE, which allows the user to input the fraction of the genome that is predicted to be missing due to incompleteness (and to correct gene loss rates accordingly). We estimated completeness for each genome using CheckM. We have now clarified this in the methods (ln 518-520).

Minor comments:
I suggest to label the three of the six known families that have the first genomes in the Fig. 1 tree. Meanwhile, it will also be better to label the Nitrososphaerales clade in the tree.
The Nitrososphaerales clade has been highlighted by an arrow, the new genomes are indicated in red font and the three families for which the new genomes are the first representatives are indicated with dotted red branches.

Please clarify the importance of the newly discovered 12 MAGs in assisting the whole phylum level evolutionary analysis. The contribution of the well-established Nitrososphaerales genome collection to the evolutionary analysis should be also highlighted. Else, this seems like revisiting Thaumarchaeota evolutionary histories.
The new supplementary figure (Fig S1) now clearly indicates the phylogenetic importance of the newly discovered 12 MAGs. In addition, text has been added illustrating the importance of these new genomes to the analysis (ln 412-415).

Gene duplication is mentioned in the context of increasing gene numbers and genome size in each genome, that makes sense. I however, read proteome expansion to imply functional expansion. But is this true? I think clarifying this one way or another would be useful

We have now added a section in the discussion describing how gene duplication can lead to functional expansion (ln 422-427).

Line 274, when new genes are gained, you do not know whether it is from inter-LGT or de novo gene formation. So gene origination doesn't equal to LGT.
This is correct. Originations could be inter-LGT or de novo gene formation. Here we define a inter-LGT as an originating gene family with a homologs in a database of non-Thaumarchaeota genomes. This is now explicitly stated in the Materials and Methods (ln 559-561).

Line 277, do you mean that if these gene families have homologs in larger databases, they are more likely to be from LGT events? But how about it is due to its LGT to other groups?
LGT to other groups is of course a possibility and these results can only be said to indicate LGT and not conclusively prove it. This and all other attempts to identify the true origins of gene families are only predictions and should be treated with appropriate scepticism. With this in mind, however, we have replaced the word "probably" with "predicted to have been" on line 302 and as stated in the last comment we have explicitly stated our criteria from predicting Intra-phyla LGT in the Materials and Methods.

Overall, I suggest the authors to label each figures to make them easier to follow. They are not particularly reader friendly.
We have amended all figures with further labelling and also carefully revised the legends

Technical problems:
Line 132. The period is not necessary.
This has been removed
Line 152-154. Add citation to the early-acquired traits.
Table S7, which contains arCOG annotations to the early-acquired genes, has now been cited (ln 182).

Reviewer #1 (Remarks to the Author):

The authors have addressed all my comments and I'm satisfied with their responses.

Reviewer #2 (Remarks to the Author):

The authors did a very good job addressing the reviewers' comments, particularly in framing their hypotheses and relevance of the results in a broader evolutionary context, as well as in consolidating nomenclature and taxonomy with previous literature. I believe the story and outcome of the study are much clearer now.

I have, however, a few additional minor comments:

Abstract lines 20-22: This is a bit misleading, as it is unclear why genomes from that particular underrepresented lineage are necessary to study the evolution of all Thaumarchaeota - especially since all results in the abstract refer to Nitrososphaerales. As I see it, this is a comparative genomics study of the whole phylum, but with particular focus on Nitrososphaerales. I understand that the abstract length is limited, but I suggest mentioning that the study was based on a large set of currently available thaumarchaeotal genomes, complemented with new genomes of the under-sampled lineage of particular interested here.

Line 40: Mesophilic is a property of an organism, not of soils, which would rather be "temperate". In any case, AOA are widespread pretty much in every soil, including in the poles and in hot deserts, so I would just remove "mesophilic.

Line 101: The manuscript is proposing a "taxonomic ranking", rather than a classification. A classification would imply a nomenclature, which is outside the scope of the study.

Line 104: Please specify here how many classes, orders, etc, correspond to AOA and/or non-AOA. Since the following results on phylogenomics/taxonomy, and most of the study, are focused on AOA, the reader is misled to think that most of this diversity indeed represents AOA. It is best to clarify that at the beginning before getting into further detail. The high taxonomic diversity of non-AOA is a remarkable result, so it is fine to highlight that both here and later when mentioned in relation to the number of genomes available.

Line 147: I assume that "g_EN76" refers to N. viennensis, whose observed OGT in culture is actually 42°C (Stieglmeier et al. 2014, IJSEM), despite being isolated from a temperate soil. This appears to agree with the predicted OGT, and deserves being mentioned. Note also that N. gargensis, which is considered a thermophile, grows optimally in culture at just 4°C above that temperature.

Lines 169-171: This statement is not entirely accurate: a Nitrososphaerales-only phylogenomic tree with only 22 leaves cannot be directly compared with phylogenies of whole amoA diversity, including far greater diversity within Nitrososphaerales alone. One cannot tell from the text if reconstructing an amoA tree with only the same level of diversity would actually yield the same topology. Likewise, one cannot tell if the current topology resulted from a reduced diversity, excluding all the putative intermediate branches, and a disproportionally high amount of phylogenetic information per leave. A model with a positive outcome is not necessarily the correct model! As it is unclear if the phylogenomic-informed rooting was what made a difference, it is incorrect to imply that this could have not been achieved with the amoA gene alone. The approach used here is perfectly fine, but the result would be reported more accurately along the lines "…we used this approach to resolve relationships among Nitrososphaerales and obtained a robust topology not observed previously in full amoA phylogenies…". I suggest adding also an acknowledgement of potential biases due to limited diversity coverage, compared to known amoA diversity, despite the much greater statistical power of

the phylogenomic data.

Lines 299-300: Please make references to branches/LCAs consistent throughout the manuscript. Here, only branch numbers are used, whereas in other parts and in Fig. 3e, only their corresponding LCA designations, and in Fig. 3b, branch NS-2 LCA/280 is also referred to as branch C.

Reviewer #3 (Remarks to the Author):

I have seen this revised manuscript by Sheridan et al and I am happy that the authors have toned down some the claims and made extensive revisions that in my opinion have very much improved the manuscript. Overall, the manuscript reads well and will represent an important contribution to the field – there are only a few remaining suggestions.

While I think more genomes will increase confidence in the findings, I can understand that acquiring these may not be possible.

That being said, I still think 45% genome completeness used for ALE is low and the authors reply did not address one specific concern. I understand that ALE incorporates measure of genome completeness into the analyses but why choose 45% as a cutoff? Is it because this was the lowest genome completion in this dataset or is there a more nuanced reason? As an example – would it be okay to use genomes that were 20% complete? Adding some clarification in this regard would be important.

Line 238-241: I would suggest explicitly saying here that this is a hypothesis.

**Response to REVIEWERS' COMMENTS**

Reviewer #1 (Remarks to the Author):

The authors have addressed all my comments and I'm satisfied with their responses.

Reviewer #2 (Remarks to the Author):

The authors did a very good job addressing the reviewers' comments, particularly in framing their hypotheses and relevance of the results in a broader evolutionary context, as well as in consolidating nomenclature and taxonomy with previous literature. I believe the story and outcome of the study are much clearer now.

I have, however, a few additional minor comments:

Abstract lines 20-22: This is a bit misleading, as it is unclear why genomes from that particular underrepresented lineage are necessary to study the evolution of all Thaumarchaeota - especially since all results in the abstract refer to Nitrososphaerales. As I see it, this is a comparative genomics study of the whole phylum, but with particular focus on Nitrososphaerales. I understand that the abstract length is limited, but I suggest mentioning that the study was based on a large set of currently available thaumarchaeotal genomes, complemented with new genomes of the under-sampled lineage of particular interested here.

This has been amended.

Line 40: Mesophilic is a property of an organism, not of soils, which would rather be "temperate". In any case, AOA are widespread pretty much in every soil, including in the poles and in hot deserts, so I would just remove "mesophilic.

This has been removed.

Line 101: The manuscript is proposing a "taxonomic ranking", rather than a classification. A classification would imply a nomenclature, which is outside the scope of the study.

This has been amended.

Line 104: Please specify here how many classes, orders, etc, correspond to AOA and/or non-AOA. Since the following results on phylogenomics/taxonomy, and most of the study, are focused on AOA, the reader is misled to think that most of this diversity indeed represents AOA. It is best to clarify that at the beginning before getting into further detail. The high taxonomic diversity of non-AOA is a remarkable result, so it is fine to highlight that both here and later when mentioned in relation to the number of genomes available.

This information has been added.

Line 147: I assume that "g_EN76" refers to N. viennensis, whose observed OGT in culture is actually 42°C (Stieglmeier et al. 2014, IJSEM), despite being isolated from a temperate soil. This appears to

agree with the predicted OGT, and deserves being mentioned. Note also that N. gargensis, which is considered a thermophile, grows optimally in culture at just 4°C above that temperature.

Comparisons between *in silico* and *in vitro* predictions of optimal growth temperatures have been explored in ‘SI: Extended optimal growth temperature’. This SI has now been mentioned in the sentence as well.

Lines 169-171: This statement is not entirely accurate: a Nitrososphaerales-only phylogenomic tree with only 22 leaves cannot be directly compared with phylogenies of whole amoA diversity, including far greater diversity within Nitrososphaerales alone. One cannot tell from the text if reconstructing an amoA tree with only the same level of diversity would actually yield the same topology. Likewise, one cannot tell if the current topology resulted from a reduced diversity, excluding all the putative intermediate branches, and a disproportionally high amount of phylogenetic information per leave. A model with a positive outcome is not necessarily the correct model! As it is unclear if the phylogenomic-informed rooting was what made a difference, it is incorrect to imply that this could have not been achieved with the amoA gene alone. The approach used here is perfectly fine, but the result would be reported more accurately along the lines "…we used this approach to resolve
relationships among Nitrososphaerales and obtained a robust topology not observed previously in full amoA phylogenies**…". I suggest adding also an acknowledgement of potential biases due to limited diversity coverage, compared to known amoA diversity, despite the much greater statistical power of the phylogenomic data.**

This has been amended.

Lines 299-300: Please make references to branches/LCAs consistent throughout the manuscript. Here, only branch numbers are used, whereas in other parts and in Fig. 3e, only their corresponding LCA designations, and in Fig. 3b, branch NS-2 LCA/280 is also referred to as branch C.
This has been amended and a note explaining that the extensive duplication C happened in NS-2 LCA has been added into the figure legend.

Reviewer #3 (Remarks to the Author):

I have seen this revised manuscript by Sheridan et al and I am happy that the authors have toned down some the claims and made extensive revisions that in my opinion have very much improved the manuscript. Overall, the manuscript reads well and will represent an important contribution to the field – there are only a few remaining suggestions.

While I think more genomes will increase confidence in the findings, I can understand that acquiring these may not be possible.

That being said, I still think 45% genome completeness used for ALE is low and the authors reply did not address one specific concern. I understand that ALE incorporates measure of genome completeness into the analyses but why choose 45% as a cutoff? Is it because this was the lowest genome completion in this dataset or is there a more nuanced reason? As an example – would it be okay to use genomes that were 20% complete? Adding some clarification in this regard would be important.

The choice of the 45% cutoff was based on a balance between genome completeness and taxonomic coverage. Our initial thought was to use a slightly lower completeness (40% completeness, as performed in a recent study on thaumarchaeotal genomes (https://doi.org/10.1038/s41396-019-0418-8)). However, investigation of the existing genomes in the database highlighted only two genomes of potential interest (without other closely-related representative with higher completeness) between 40 and 50% completeness: ARK01 (49.78% completeness; unique representative of a non-AOA class) and 13-1-40CM-48-12 (48.06% completeness; a genome from the f_Nitrososphaeraceae family), guiding our choice towards the 45% completeness.

We are confident that 45 % cutoff is appropriate for our specific dataset, as the majority of low completeness genomes are in well represented NP clades, mitigating any missing data effects on proximal branches, but this cutoff may not be appropriate to all datasets.

We have added text in the Methods section to emphasise these two points.

Line 238-241: I would suggest explicitly saying here that this is a hypothesis.

This has been amended.
